# Advancing drug-drug interactions research: integrating AI-powered prediction, vulnerable populations, and regulatory insights

**DOI:** 10.3389/fphar.2025.1618701

**Published:** 2025-08-13

**Authors:** Wenzhun Huang, Xiao Wang, Yunhao Chen, Changqing Yu, Shanwen Zhang

**Affiliations:** ^1^ School of Electronic Information, Xijing University, Xi’an, Shaanxi, China; ^2^ Key Laboratory of High Precision Industrial Intelligent Vision Measurement Technology, Xi’an, Shaanxi, China; ^3^ School of Intelligent Manufacturing and Control Engineering, Qilu Institute of Technology, Jinan, China; ^4^ Shandong Provincial Key Laboratory of Industrial Big Data and Intelligent Manufacturing, Qilu Institute of Technology, Jinan, China

**Keywords:** drug-drug interactions, artificial intelligence, clinical decision support systems, polypharmacy, pharmacogenomics, knowledge graphs, vulnerable populations, regulatory science

## Abstract

Drug-drug interactions (DDIs) pose a significant and intricate challenge in clinical pharmacotherapy, especially among older adults who often have chronic conditions that necessitate multiple medications. These interactions can undermine the effectiveness of treatments or lead to adverse drug reactions (ADRs), which in turn can increase illness rates and strain healthcare resources. Traditional methods for detecting DDIs, such as clinical trials and spontaneous reporting systems, tend to be retrospective and frequently fall short in identifying rare, population-specific, or complex DDIs. However, recent advancements in artificial intelligence (AI), systems pharmacology, and real-world data analytics have paved the way for more proactive and integrated strategies for predicting DDIs. Innovative techniques like graph neural networks (GNNs), natural language processing, and knowledge graph modeling are being increasingly utilized in clinical decision support systems (CDSS) to improve the detection, interpretation, and prevention of DDIs across various patient demographics. This review aims to provide a thorough overview of the latest trends and future directions in DDIs research, structured around five main areas: (1) epidemiological trends and high-risk drug combinations, (2) mechanistic classification of DDIs, (3) methodologies for detection and prediction, particularly those driven by AI, (4) considerations for vulnerable populations, and (5) regulatory frameworks and pathways for innovation. Special emphasis is placed on the role of pharmacogenomic insights and real-world evidence in developing personalized strategies for assessing DDIs risks. By connecting fundamental pharmacological principles with advanced computational technologies, this review seeks to guide clinicians, researchers, and regulatory bodies. The integration of AI, multi-omics data, and digital health systems has the potential to significantly enhance the safety, accuracy, and scalability of DDIs management in contemporary healthcare.

## Highlights

- This article provides a comprehensive overview of the latest advancements in drug-drug interactions (DDIs) research, highlighting the integration of an analytical framework that combines artificial intelligence, knowledge graphs, and clinical decision support systems for predicting DDIs.
- It underscores the importance of understanding DDI risks and implementing effective management strategies, particularly for vulnerable populations such as older adults, pregnant women, and children.
- The article also compares various international regulatory approaches and classification systems that are pertinent to DDIs assessment, offering insights into how different regions address these challenges.
- Furthermore, it identifies key future research priorities, including the need for model interpretability, the development of personalized risk alerts, and the integration of pharmacogenomics into DDIs studies.
- The emphasis is placed on a convergence-oriented perspective of DDIs risk assessment, which seeks to bridge the fields of clinical pharmacology, machine learning, and regulatory translation, ultimately aiming to enhance real-world applications in the management of drug interactions.

## 1 Introduction

### 1.1 Challenges of DDIs and traditional methods

Drug-drug interactions (DDIs) present a significant challenge in pharmacotherapy, a situation that is becoming more complex due to the aging global population and the increasing rates of chronic multimorbidity. DDIs arise when two or more drugs taken together influence each other’s pharmacokinetic or pharmacodynamic properties. This interaction can lead to a decrease in therapeutic effectiveness, unexpected side effects, or even severe, life-threatening consequences. The issue is particularly pronounced with the rise of polypharmacy, especially in elderly individuals and hospitalized patients, which has drawn increased attention from clinicians, researchers, and regulatory agencies focused on understanding and managing these interactions effectively.

### 1.2 Limitations of traditional DDI detection methods

Traditionally, identifying DDIs has depended on retrospective methods, including clinical observations, post-marketing surveillance, and spontaneous reporting of adverse events. Although these approaches provide valuable insights, they often suffer from fragmentation and lack the sensitivity and timeliness needed for proactive pharmacovigilance. Consequently, many DDIs go undetected, leading to preventable adverse drug events and adding strain to healthcare systems. Alarmingly, around 30% of adverse drug reactions (ADRs) are associated with DDIs, with a considerable number of these interactions remaining unrecognized in clinical practice.

### 1.3 The role of emerging technologies in DDI detection

In recent years, significant advancements in systems biology, pharmacokinetics, and molecular pharmacology, along with the emergence of artificial intelligence (AI), machine learning (ML), and network pharmacology, have transformed DDIs research. These innovative technologies facilitate the large-scale prediction and mechanistic investigation of potential DDIs, frequently uncovering risks before they become apparent in clinical settings.

### 1.4 Gaps and challenges in current research

Despite the promising advancements in the field, there are still notable gaps that need to be addressed. Many current reviews tend to overlook recent developments in computational methods, as well as the valuable real-world data derived from electronic health records (EHRs). Additionally, they often fail to consider the specific DDIs risks that vulnerable populations, such as the elderly and critically ill patients, face. This highlights the urgent need for a comprehensive and up-to-date synthesis that tackles these challenges effectively. While tools like the STOPP/START criteria are increasingly being utilized to minimize potentially inappropriate medications (PIMs) and DDIs events in geriatric care, further research is essential to fully understand and address the wide range of DDIs risks present in these populations.

### 1.5 The framework and objectives of this review

This review presents a comprehensive synthesis of the current understanding of DDIs, covering epidemiological trends, mechanistic insights, predictive methodologies, and regulatory perspectives. It emphasizes the impact of emerging technologies, particularly artificial intelligence (AI) and pharmacogenomics, on the detection and prevention of DDIs. Unlike earlier reviews that typically focus on specific mechanistic or computational elements, this work combines AI, pharmacogenomics, and regulatory science into a cohesive framework, providing a more expansive view of DDIs research.

### 1.6 The proposed multidimensional framework

**FIGURE 1:**
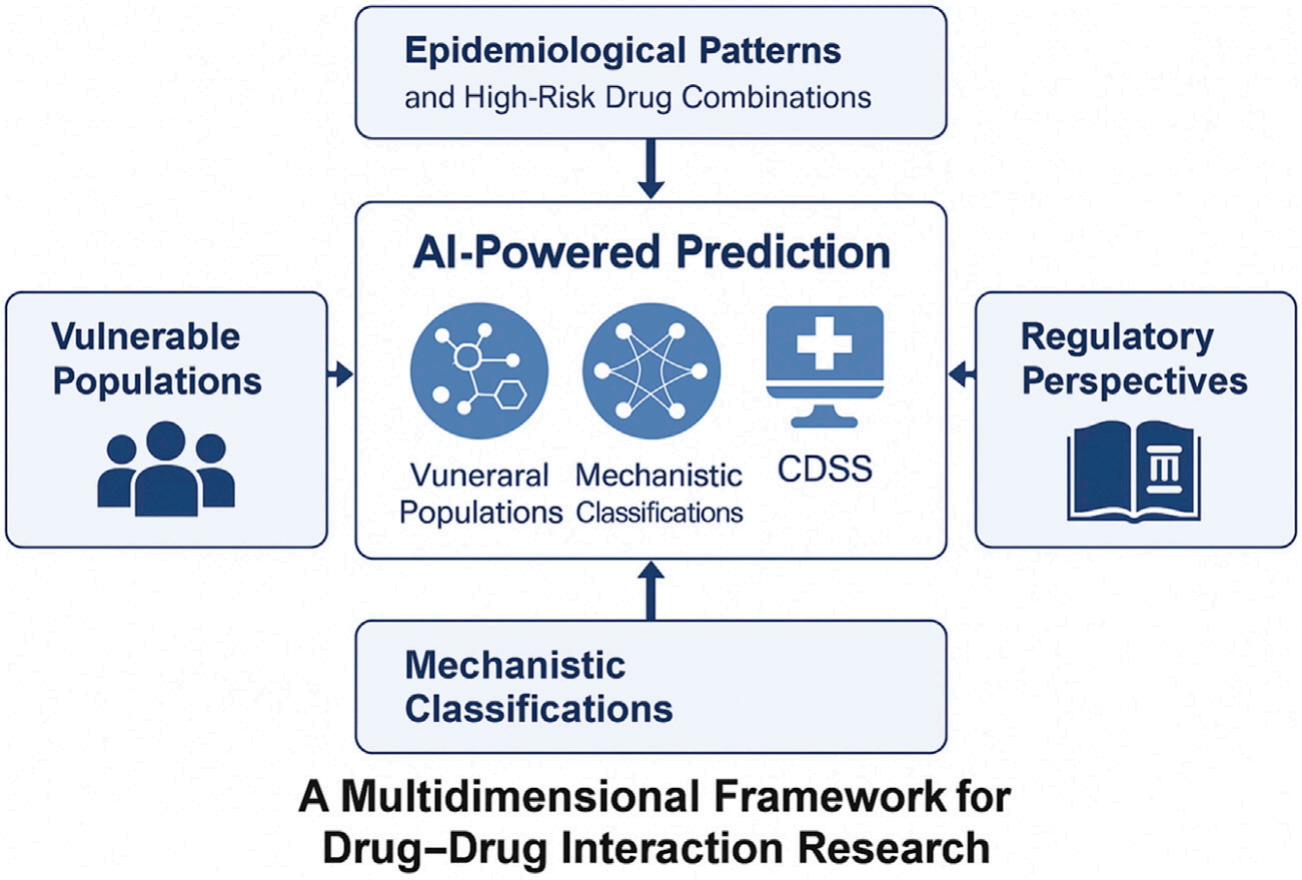
A multidimensional framework for contemporary drug–drug interaction (DDI) research. The figure depicts five key thematic pillars in modern DDI research: epidemiological patterns, mechanistic classifications, AI-based prediction strategies, vulnerable populations, and regulatory strategies. Artificial intelligence acts as a central integrator across these domains, bridging pharmacogenomics, real-world data, and knowledge graph modeling to support proactive and personalized DDI risk management.

To assist readers in navigating this dynamic landscape, we present a comprehensive framework (Figure 1) that encompasses five essential components: epidemiological patterns, mechanistic classifications, AI-driven prediction methodologies, risk factors affecting vulnerable populations, and regulatory strategies. Central to this framework is artificial intelligence (AI), which serves as a crucial link connecting fundamental scientific research, clinical applications, and health policy.

This integrative approach highlights the crucial role of combining pharmacological knowledge with data-driven innovation to effectively shape the future of DDIs management.

## 2 Epidemiological landscape of DDIs

DDIs have emerged as a significant concern in modern pharmacotherapy, especially given the aging population and the increasing incidence of multimorbidity. Research shows that DDIs are a major contributor to adverse drug reactions (ADRs), which can lead to more hospital admissions, longer treatment times, and higher healthcare costs. This section outlines the current state of epidemiological research on DDIs, focusing on trends in prevalence, populations at higher risk, frequently involved drug combinations, and findings from real-world pharmacovigilance systems. The use of multiple medications, or polypharmacy, along with inappropriate prescribing practices, greatly increases the likelihood of clinically significant DDIs among older adults. Tools like the STOPP/START criteria have proven effective in minimizing the use of potentially inappropriate medications, as highlighted by a study conducted by Güvel et al. (2024) involving geriatric patients in Turkey.

Recent studies have employed data mining techniques to identify significant DDIs within common drug combinations used to manage chronic conditions such as diabetes. For instance, research conducted by Dwivedi et al. (2025) indicates that the risk of DDIs is particularly elevated in polypharmacy situations, especially concerning anti-diabetic medications. A notable example is the concurrent use of metformin with iodinated contrast media, which significantly heightens the risk of lactic acidosis. Similarly, combining nonsteroidal anti-inflammatory drugs (NSAIDs) with sulfonylureas increases the likelihood of hypoglycemia. These findings highlight the urgent need for careful monitoring and personalized treatment plans to mitigate DDIs-related risks, especially in vulnerable populations such as the elderly and individuals with multiple comorbidities.

### 2.1 Polypharmacy and DDIs risk

#### 2.1.1 Polypharmacy and DDIs Risk in general

Polypharmacy, which refers to the use of five or more medications at the same time, greatly heightens the risk of DDIs due to the complex ways in which different drugs can affect each other. These interactions can manifest through various mechanisms, such as the inhibition of enzymes that metabolize drugs, changes in how drugs are processed in the body, or even through combined effects that can amplify each drug’s impact. This situation becomes particularly challenging for patients who have multiple health issues, as their treatment plans can become more complicated. The risk of DDIs is especially significant among psychiatric inpatients, where the burden of taking many medications can lead to increased chances of harmful interactions (Wolff et al., 2021). A study conducted by Uskur et al. (2024) highlights the complications associated with lithium treatment, emphasizing the importance of closely monitoring blood levels to prevent adverse drug-drug interactions. This careful oversight is essential for effectively managing psychiatric patients who are on polypharmacy regimens.

#### 2.1.2 Polypharmacy in elderly populations

Polypharmacy is particularly common among elderly individuals, especially those dealing with several chronic conditions, and it poses significant risks for DDIs (Hire and Franklin, 2024). For instance, Kaur et al. (2024) found that older adults receiving outpatient care often face drug-related issues stemming from polypharmacy. In a similar vein, Hart et al. pointed out that inappropriate prescribing remains a recurring challenge in this demographic, closely linked to the occurrence of DDIs (Hart et al., 2025). Together, these findings highlight that polypharmacy is not only widespread but also a serious concern within aging populations.

#### 2.1.3 DDI risks in specific subgroups

A deeper concern emerges when we look closely at specific subgroups, particularly elderly patients with neurocognitive disorders. These individuals are especially susceptible to polypharmacy, particularly with anticholinergic medications, which can worsen cognitive decline (Perdixi et al., 2024). Brown et al. (2021) highlight the issue of care fragmentation in dementia patients, which complicates the management of their medications (Kern et al., 2024). Additionally, elderly cancer patients receiving chemotherapy face an increased risk of DDIs due to polypharmacy, as demonstrated by Oliveira et al. (2024).

Polypharmacy and alcohol use frequently occur together in individuals living with HIV, which heightens the risk of DDIs and can lead to decreased adherence to prescribed treatments (Womack et al., 2025). This issue is not limited to HIV-positive patients; individuals with psychiatric disorders, especially those taking medications like clozapine or lithium, also face significant concerns regarding DDIs (Verdoux et al., 2024a; Ruan et al., 2024; Verdoux et al., 2024b; Baptista et al., 2024). Research by Zhang et al. (2024) highlighted that the pharmacokinetics of aripiprazole in patients with schizophrenia are particularly affected by polypharmacy. These findings indicate that both older adults and populations with psychiatric or immunocompromised conditions are at an increased risk for experiencing harmful DDIs.

#### 2.1.4 Efforts to mitigate DDIs risk: AI and deprescribing strategies

Efforts to mitigate the risk of DDIs are increasingly utilizing artificial intelligence (AI) and medication management tools. For example, AI-driven platforms, including chatbots, can assist healthcare providers in making real-time decisions by addressing common inquiries related to medications (Albogami et al., 2024). Strategies for medication review and deprescribing, as highlighted by researchers Carollo et al. and Almodovar et al., have proven effective in minimizing DDIs risks and enhancing therapeutic outcomes (Carollo et al., 2024; Almodovar et al., 2024). A significant development in this area is the introduction of long-acting injectable antiretroviral therapy (LAI ART), which presents new DDIs challenges, especially when combined with antibiotics or antiviral medications (Stout et al., 2024). Additionally, the emergence of new drugs, such as cytisine for smoking cessation, necessitates careful monitoring for potential interactions, particularly among hospitalized patients (Torazzi et al., 2024).

#### 2.1.5 Current gaps and future directions

Despite recent advancements in medication management, significant gaps still exist. Surveys reveal that more than one-third of individuals aged 65 and older are prescribed five or more medications daily, with this figure exceeding 50% among those with chronic conditions. The study by Alemayehu et al. (2024) found that the occurrence of potential drug-drug interactions (pDDIs) is a serious global issue, with an overall prevalence of 50.69% among elderly patients, and moderate interactions being the most common. Similarly, research by Wolff et al. (2021) highlighted a high incidence of clinically significant DDIs in psychiatric settings. The drug classes most commonly involved in these interactions included antipsychotics, antihypertensives, and anticoagulants. Contributing factors to these issues include limited pharmacist-led DDIs assessments (Alhussain et al., 2024), decreased medication literacy at the time of discharge (Mubaslat et al., 2024), and a lack of vigilance among frail elderly individuals or those who consume alcohol (Gentile et al., 2024; Zare et al., 2024; Inglis et al., 2024; Wu et al., 2024). In institutional settings, the use of potentially inappropriate medications (PIMs) continues to be a significant factor leading to adverse DDIs (Andrade et al., 2024).

#### 2.1.6 Conclusion and future research

In summary, polypharmacy is a significant factor contributing to the risk of DDIs, especially in vulnerable groups like the elderly, psychiatric patients, and individuals with complicated treatment plans. Although artificial intelligence (AI) technologies and deprescribing strategies show potential in addressing these issues, ongoing efforts are essential to effectively incorporate these solutions into various clinical settings. Future research should focus on the practical application of AI, interventions led by pharmacists, and monitoring DDIs tailored to specific regions to thoroughly reduce these risks. A comparative overview of DDIs risks across different populations is provided in Table 1.

**TABLE 1:** Comparison of drug–drug interaction (DDI) risk among vulnerable populations.

Vulnerable population	Common medication types involved	Key DDI risk factors	Representative studies (ref. No.)
Elderly patients (≥65 years)	Anticholinergics, Antihypertensives, Psychotropics	Polypharmacy, frailty, PIMs, cognitive decline	Uskur et al. (2024), Kaur et al. (2024), Hart et al. (2025), Perdixi et al. (2024), Kern et al. (2024), Albogami et al. (2024), Carollo et al. (2024), Zare et al. (2024), Wu et al. (2024)
Psychiatric inpatients	Antipsychotics (e.g., clozapine, lithium, aripiprazole)	Polypharmacy, narrow therapeutic index drugs, poor adherence	Dwivedi et al. (2025), Womack et al. (2025), Verdoux et al. (2024a), Ruan et al. (2024), Verdoux et al. (2024b), Baptista et al. (2024)
Patients with HIV	Antiretrovirals, Antibiotics, Alcohol use	Drug–alcohol interactions, LAI ART–induced DDIs	Oliveira et al. (2024), Almodovar et al. (2024)
Cancer patients (elderly)	Chemotherapeutics, Supportive meds	Intensive treatment regimens, polypharmacy	Kern et al. (2024)
Dementia patients	Anticholinergics, Psychotropics	Poor care coordination, cognitive vulnerability	Hart et al. (2025), Perdixi et al. (2024)
Hospitalized older adults	Multiclass drugs, Cytisine, Cardiovascular agents	Frequent regimen changes, inadequate discharge education	Stout et al. (2024), Alhussain et al. (2024), Mubaslat et al. (2024)
Hypertensive patients	Antihypertensives, NSAIDs	Chronic disease management, lack of DDI monitoring	Gentile et al. (2024)
Patients with multiple prescribers	Mixed drug classes	Inconsistent medication review, communication gaps	Carollo et al. (2024), Torazzi et al. (2024)

### 2.2 DDIs prevalence in vulnerable populations

Certain populations experience a higher incidence of DDIs. A retrospective cohort study published in Frontiers in Pharmacology by Schneider et al. (2021) revealed that older adults suffering from comorbid conditions such as hypertension, diabetes, and depression were prescribed an average of eight medications each day. This polypharmacy resulted in 3 to 5 potentially dangerous drug-drug interactions (pDDIs) per patient. Alarmingly, more than 60% of these interactions were deemed clinically significant, posing risks such as bleeding events or reduced therapeutic effectiveness. Thus, polypharmacy presents a complex challenge in geriatric care, where the goal of effective treatment must be carefully weighed against the potential for harmful interactions (Swinglehurst et al., 2023).

The prevalence of potentially dangerous drug interactions (pDDIs) is notably higher in intensive care units (ICUs) and long-term care facilities. A population-based study published in the Journal of the American Medical Directors Association by Anrys et al. (2021) found that 77.3% of elderly nursing home residents experienced at least one pDDIs, with 18.5% categorized as high-risk. Such interactions can lead to serious consequences, including delirium, hemorrhage, and acute kidney injury.

### 2.3 High-risk drug combinations in clinical settings

Real-world evidence has consistently highlighted certain drug combinations as particularly dangerous. Among the notable high-risk DDIs are:

• **Warfarin + NSAIDs**: Elevates bleeding risk through both pharmacokinetic (CYP inhibition) and pharmacodynamic (antiplatelet) mechanisms.
• **SSRIs + TCAs**: Increases the likelihood of central nervous system (CNS) adverse effects, including sedation and serotonin syndrome.
• **ACEIs/ARBs + Diuretics + NSAIDs (“triple whammy”)**: Raises the risk of acute kidney injury via additive nephrotoxic effects and reduced glomerular filtration.
• **Antiepileptics + Antiretrovirals**: Compete for metabolic enzymes, potentially altering the efficacy and toxicity of both agents.

These interactions often appear gradually and can manifest in various ways, including elevated liver enzymes, subtle changes in neurological function, or a diminished response to treatment. This makes it challenging for clinicians to detect these issues without the use of proactive screening tools.

**FIGURE 2:**
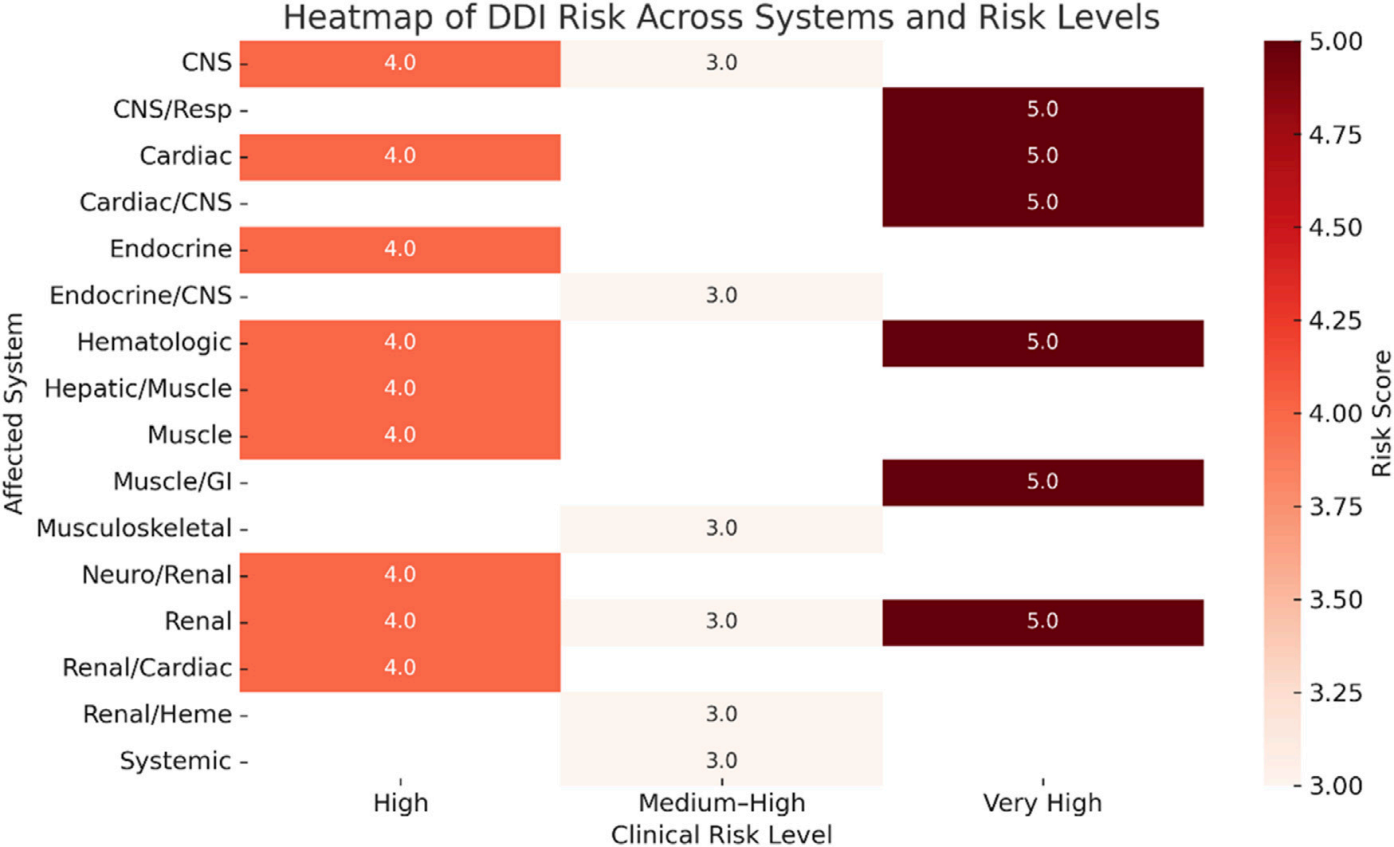
Heatmap of drug–drug interaction (DDI) risk scores across clinical systems and risk levels.

Figure 2 illustrates the average DDIs risk scores, measured on a scale from 3 to 5, across various organ systems, categorized by clinical risk levels: “Medium-High,” “High,” and “Very High.” The use of darker shades indicates higher average risk scores. It is particularly noteworthy that the renal and cardiovascular systems are consistently associated with very high-risk interactions, highlighting the critical need for careful monitoring in these areas.

**FIGURE 3:**
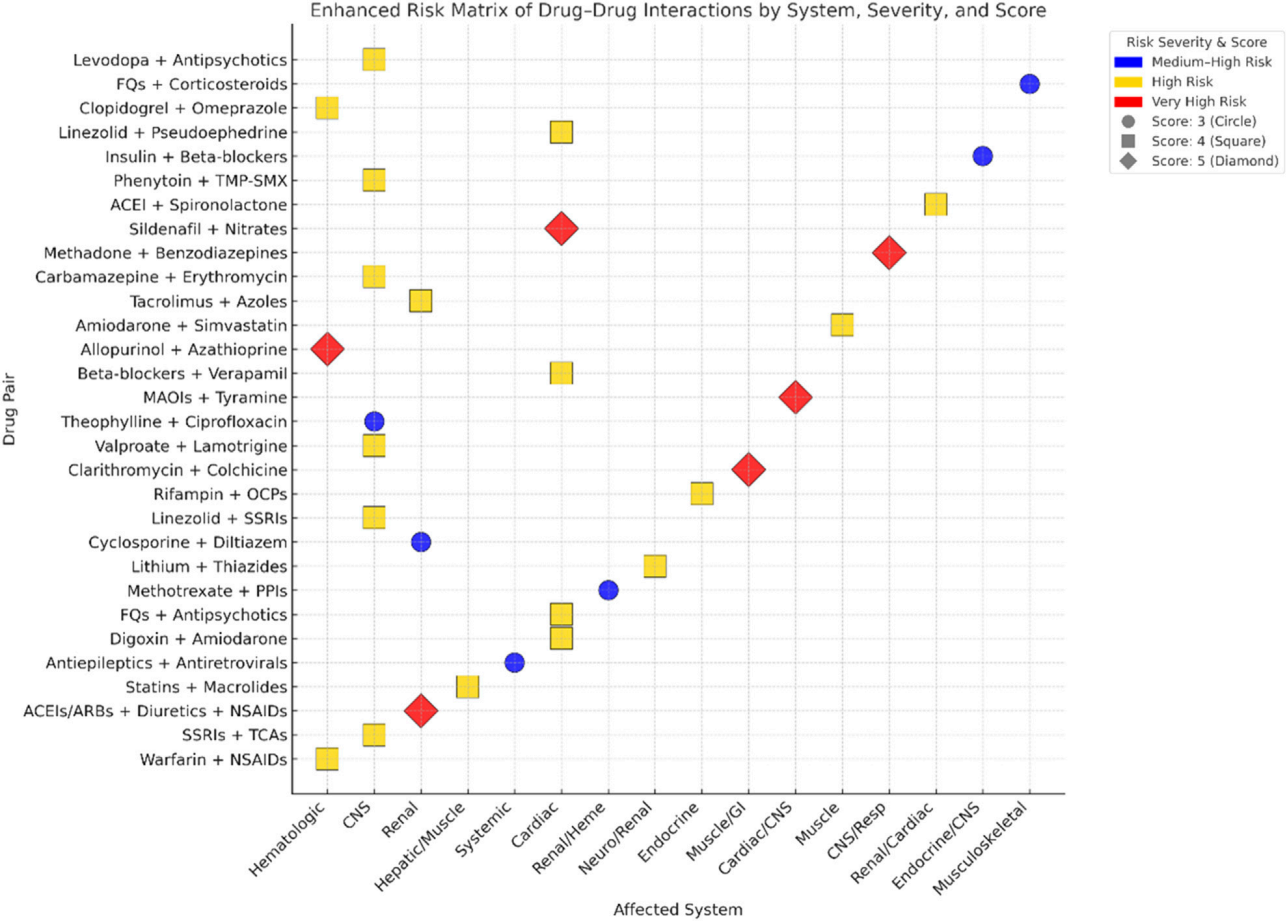
Enhanced matrix of high-risk drug–drug interactions by system, severity, and risk score.

Figure 3 presents a two-dimensional mapping of 30 high-risk DDIs pairs. In this visualization, drug combinations are plotted along the X-axis, which represents the affected physiological system, and the Y-axis, which denotes the identity of the drugs involved. The color coding indicates the clinical risk level associated with each interaction, with blue representing medium-high risk, gold indicating high risk, and red signifying very high risk. Additionally, the shape and size of the points on the graph correspond to the risk score of each interaction, where circles represent a score of 3, squares indicate a score of 4, and diamonds denote a score of 5. This graphical representation allows for the quick identification of priority interactions across various physiological systems, enhancing the ability to assess and manage potential risks in clinical settings.

### 2.4 Insights from pharmacovigilance databases

Large-scale pharmacovigilance platforms, including the FDA Adverse Event Reporting System (FAERS), EudraVigilance, and institutional electronic health records (EHRs), offer crucial epidemiological insights into DDIs. Analyses of data from FAERS indicate that approximately 12%–15% of serious adverse drug reactions (ADRs) could be linked to either known or previously unrecognized DDIs. However, several challenges persist in this area, such as underreporting of adverse events and the absence of standardized taxonomies for categorizing DDIs.

Recent efforts utilizing artificial intelligence (AI) and natural language processing (NLP) to analyze real-world data (RWD) have uncovered the often-overlooked burden of DDIs. These advanced tools are capable of extracting structured signals of interactions from unstructured clinical notes, which frequently leads to the discovery of clinically significant interactions that are not recorded in traditional reference databases. Additionally, studies based on electronic health records (EHR) have emphasized the existence of population-specific DDIs profiles, particularly within pediatric, pregnant, and geriatric groups.

### 2.5 Under-recognition and clinical implications

Despite growing awareness, many DDIs continue to go unrecognized, often due to factors such as delayed onset, nonspecific clinical symptoms, or a lack of familiarity among clinicians. For example, interactions involving warfarin may first appear as minor bruising, which can later escalate to more severe complications like gastrointestinal or intracranial hemorrhage. In a similar vein, interactions affecting the central nervous system (CNS) frequently present with symptoms like fatigue or cognitive changes, which may be mistakenly attributed to the natural progression of an underlying disease rather than the effects of medications.

The consequences of missed DDIs can be quite serious. Research indicates that as many as 30% of hospitalizations related to adverse drug reactions (ADRs) could be avoided with proper monitoring of DDIs. When these interactions go undetected, it can lead to ineffective treatment, higher rates of illness, and, in certain instances, even death.

### 2.6 The need for early detection and intervention

Given the increasing frequency and seriousness of DDIs, it is essential to adopt proactive measures for their detection. Incorporating DDIs screening into clinical practices can significantly reduce their public health implications. This can be achieved by utilizing clinical decision support systems (CDSS), conducting pharmacist-led medication reviews, and providing ongoing medical education for healthcare professionals. These strategies work together to enhance awareness and management of DDIs, ultimately improving patient safety and outcomes.

Risk stratification that incorporates demographic and clinical variables can significantly enhance precision pharmacovigilance. For instance, elderly patients who are on multiple medications, known as polypharmacy, could be automatically identified as high-risk for dangerous drug combinations through the use of machine learning-enabled CDSS. Additionally, it is essential to prioritize the creation of region-specific drug-drug interaction (DDI) surveillance tools that are customized to reflect local prescribing habits and genetic profiles. This approach will improve risk mitigation strategies by ensuring they are context-sensitive and relevant to the populations being served.

## 3 Molecular mechanisms and classifications of drug-drug interactions

**FIGURE 4:**
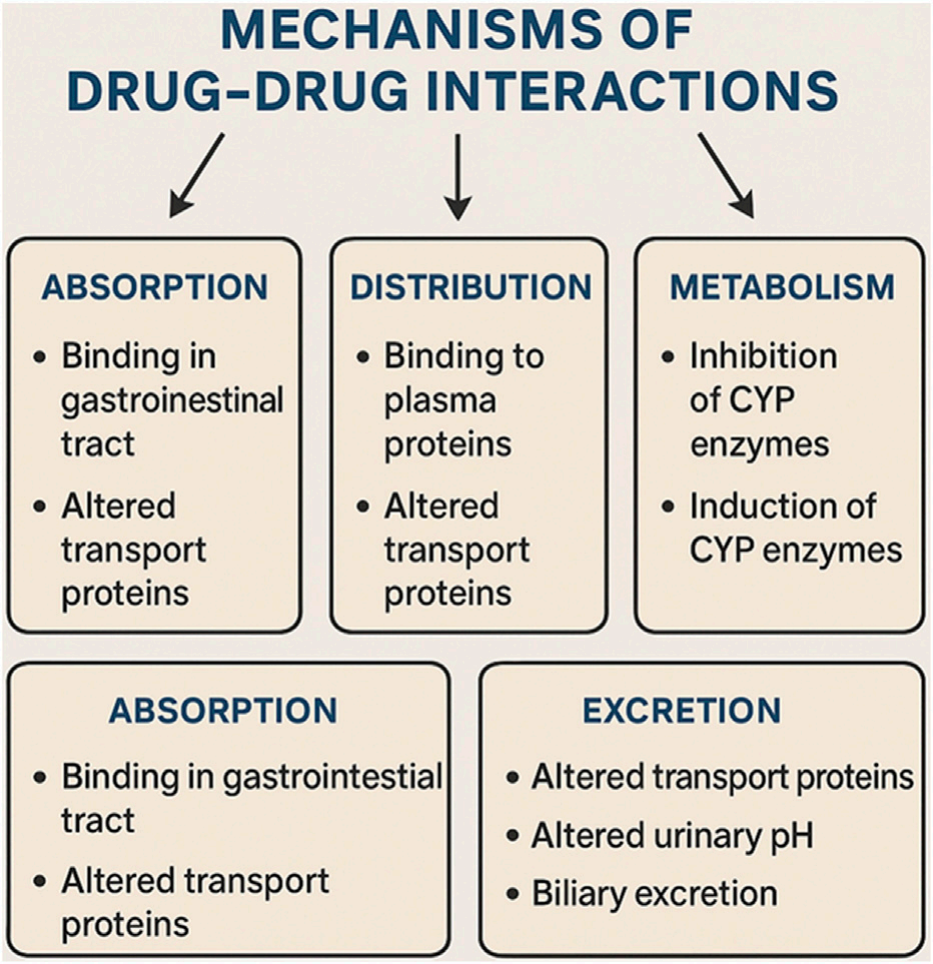
Mechanisms of drug-drug interactions.

Understanding the molecular mechanisms behind DDIs is essential for anticipating possible adverse effects and enhancing pharmacotherapy. DDIs can take place at various biological levels, such as the modulation of enzymes, interference with transporters, interactions at receptors, and changes in downstream signaling pathways. Depending on the nature of these biological interactions, DDIs are typically divided into two main categories: pharmacokinetic (PK) interactions and pharmacodynamic (PD) interactions. This classification is illustrated in Figure 4, which highlights important molecular pathways along with representative pairs of drugs involved in these interactions.

### 3.1 Pharmacokinetic interactions: Modulation of ADME processes

Pharmacokinetic DDIs occur when one medication influences the absorption, distribution, metabolism, or excretion (ADME) of another, leading to alterations in plasma drug levels and, as a result, changes in pharmacological effects. A notable example of this is lithium, which necessitates careful therapeutic monitoring because of its significant potential for interactions (Fiorillo et al., 2023).

#### 3.1.1 Absorption interactions

Absorption-related DDIs primarily take place in the gastrointestinal tract and are affected by various factors, including changes in pH, chelation, gastric motility, and modulation of transporters. For instance, proton pump inhibitors (PPIs) such as omeprazole can lower the bioavailability of certain medications like ketoconazole or atazanavir by raising the gastric pH. Similarly, polyvalent cations found in antacids, such as aluminum or magnesium salts, can bind to antibiotics like tetracyclines and fluoroquinolones, hindering their absorption. Additionally, interactions mediated by transporters play a crucial role in this area. Organic anion-transporting polypeptides (OATPs), including OATP1A2 and OATP2B1, are present in the intestinal lining and help in the uptake of drugs. Inhibitors such as cyclosporine or certain components of grapefruit juice can diminish the absorption of medications like statins by blocking OATP activity. Furthermore, fat-soluble vitamins, including vitamin D, may experience decreased gastrointestinal absorption when taken alongside bile acid sequestrants or lipase inhibitors like orlistat, likely due to disrupted micelle formation (Kupisz-Urbanska et al., 2021).

#### 3.1.2 Metabolic interactions

The most thoroughly researched mechanism of DDIs involves hepatic metabolism, particularly through the cytochrome P450 (CYP450) enzyme family. Drugs can function as substrates, inhibitors, or inducers of these enzymes. Among them, CYP3A4 is the most prevalent isoform found in both the liver and intestine, responsible for the metabolism of over 50% of all drugs available on the market. When drugs such as ritonavir, ketoconazole, or clarithromycin inhibit CYP3A4, it can result in increased plasma concentrations and heightened toxicity of co-administered substrates like midazolam or simvastatin. On the other hand, enzyme induction, as seen with rifampin or carbamazepine, speeds up drug metabolism, which may diminish therapeutic effectiveness. Importantly, DDIs are not limited to phase I metabolism; phase II enzymes, including UDP-glucuronosyltransferases (UGTs) and sulfotransferases (SULTs), also have a crucial role. For example, valproic acid can inhibit UGT-mediated glucuronidation of lamotrigine, raising the risk of skin rash and neurotoxicity.

#### 3.1.3 Distribution and protein binding

Protein-binding displacement, while not frequently a major contributor to clinically significant DDIs, can still play an important role in specific situations. For instance, medications that are tightly bound to plasma proteins, such as albumin, including warfarin and phenytoin, may be displaced by other drugs like valproic acid. This displacement results in a higher concentration of the free, active form of the drug in the bloodstream. Nevertheless, in most cases, the body compensates for this increase through enhanced clearance of the drug, unless there is an impairment in liver or kidney function, which could disrupt this balance and potentially lead to adverse effects.

#### 3.1.4 Renal and biliary excretion

Drugs that are eliminated through renal tubular secretion can compete for shared transporters, specifically organic anion transporters (OAT1 and OAT3) and organic cation transporters (OCT2). A notable example is probenecid, which inhibits the OAT1-mediated excretion of penicillin, leading to an extended half-life of the drug. Additionally, the inhibition of P-glycoprotein (P-gp), which is present in both renal and biliary epithelial cells, can significantly influence drug elimination. For instance, when P-gp inhibitors like verapamil are taken alongside digoxin, they can increase the plasma levels of digoxin, heightening the risk of toxicity. This risk is particularly concerning for drugs with narrow therapeutic indices, such as lithium and digoxin, which are more susceptible to adverse interactions. Therefore, it is crucial to carefully adjust dosages and monitor therapeutic drug levels to ensure patient safety (Parmar and Pal, 2024).

### 3.2 Pharmacodynamic interactions: target-level synergy and antagonism

Pharmacodynamic DDIs arise when two medications affect the same or interconnected physiological pathways, resulting in effects that can be additive, synergistic, or antagonistic.

#### 3.2.1 Synergistic and additive effects

Drugs with similar therapeutic effects can exhibit either additive or synergistic responses. These interactions may be beneficial or harmful, depending on the clinical context. Similarly, cannabinoids such as THC and CBD can potentiate the effects of CNS depressants, including opioids and benzodiazepines, through overlapping mechanisms at GABAergic and serotonergic synapses, increasing the risk of excessive sedation and respiratory depression. Similarly, combining antihypertensives, such as ACE inhibitors, with diuretics can enhance blood pressure reduction but may also lead to hypotension or electrolyte imbalances.

#### 3.2.2 Antagonistic interactions

Antagonistic pharmacodynamic (PD) interactions can diminish the effectiveness of one or both medications involved. For instance, nonsteroidal anti-inflammatory drugs (NSAIDs) can weaken the blood pressure-lowering effects of β-blockers or ACE inhibitors by causing the body to retain sodium and constrict blood vessels. Another example is flumazenil, which is used as an antidote for benzodiazepine overdoses; it works by blocking the effects of benzodiazepines at the GABA-A receptor. Although this antagonistic interaction is beneficial in situations of overdose, it exemplifies a classic case of DDIs at the receptor level.

#### 3.2.3 Signal pathway crosstalk and off-target effects

Pharmacodynamic interactions can occur when different drugs converge on the same downstream signaling pathways. Cannabinoids, for instance, influence various receptor systems, including CB1, CB2, 5-HT1A, and TRPV1, which leads to intricate crosstalk in signal transduction (Brown et al., 2021). This complexity can result in either additive or antagonistic interactions with serotonergic and dopaminergic medications. A notable example is the combination of selective serotonin reuptake inhibitors (SSRIs) and monoamine oxidase inhibitors (MAOIs), both of which elevate synaptic serotonin levels. When these two classes of drugs are used together, they can trigger serotonin syndrome, a serious condition marked by excessive neuromuscular and autonomic activity. Furthermore, certain medications may have off-target effects that influence the efficacy of other treatments. For example, tyrosine kinase inhibitors can interfere with immune checkpoint signaling. This means that targeted therapies, such as tyrosine kinase inhibitors, may lead to unforeseen interactions, particularly when different agents are administered in sequence (Forte et al., 2024). MAOIs are particularly concerning in this context, as they pose a high risk for pharmacodynamic drug-drug interactions, especially when taken alongside serotonergic or sympathomimetic drugs (Gillman et al., 2023).

### 3.3 Classification systems and severity grading

Various classification systems have been proposed to stratify DDIs based on severity, molecular mechanism, and clinical management recommendations:

• **Severity-based grading** (e.g., minor, moderate, major): Reflects the clinical consequences, ranging from negligible to life-threatening.
• **Evidence-based grading**: Incorporates clinical trial data, case reports, and *in vitro* studies (e.g., Micromedex, Lexicomp, Stockley’s).
• **Management-oriented categorization**: Recommends specific actions, such as dose adjustment, monitoring, or complete avoidance. The Hansten and Horn classification is a widely used system, which ranks interactions from “Class 1” (avoid combination) to “Class 5” (no interaction expected).

### 3.4 Molecular tools for DDI prediction

Emerging tools like molecular docking, enzyme phenotyping, and quantitative systems pharmacology (QSP) models offer robust frameworks for predicting DDIs at a mechanistic level. For instance, CYP phenotyping panels and probe cocktails are instrumental in assessing metabolic pathways, allowing researchers to understand how different drugs may interact within the body. Additionally, *in silico* tools such as SimCYP and GastroPlus play a crucial role by simulating drug behavior under various physiological conditions, providing insights into how drugs might perform in real-world scenarios. When these advanced technologies are combined with clinical pharmacogenomics, they significantly enhance our capacity to predict and prevent clinically significant DDIs, ultimately improving patient safety and treatment outcomes.

## 4 Detection and prediction approaches for DDIs

Effective detection and prediction of DDIs are essential for preventing adverse drug reactions (ADRs), especially in situations involving polypharmacy and complex treatment plans. Traditional methods, such as post-marketing surveillance and clinical pharmacology, continue to play a vital role; however, they are increasingly being enhanced or even replaced by computational and informatics-driven techniques. This section offers a thorough overview of both established and innovative strategies for identifying DDIs, covering clinical, *in vitro*, *in silico*, and machine learning-based approaches. A particular focus is placed on integrative, knowledge-based frameworks that align with the principles of translational pharmacology. Additionally, representative open-access databases and tools are summarized to support practical implementation and ensure reproducibility. Notably, midazolam is recognized as a gold-standard probe for assessing CYP3A activity and serves as a benchmark for evaluating DDIs in clinical pharmacology (Coroa et al., 2023).

Emerging computational strategies can be classified into four main categories: rule-based systems, traditional machine learning, deep learning, and graph-based approaches (see Table 2). Each of these methods has unique advantages and disadvantages depending on the specific application context. Rule-based systems, for instance, are commonly used in clinical environments because they are easy to interpret. However, they often suffer from high false-positive rates and can lead to clinician alert fatigue, which may undermine their practical effectiveness.

**TABLE 2:** Comparison of prediction methods for drug–drug interaction modeling, with representative open-access resources.

Method type	Representative models	Advantages	Disadvantages	Application scenarios	Representative resources
Rule-based	Decision Rules, FAERS Mining	Easy to interpret, domain knowledge-based	Poor generalization, limited scalability	Early adverse event detection	DrugBank, Medscape, Lexicomp
Traditional ML	SVM, Random Forest, XGBoost	High efficiency, interpretable	Requires manual features, prone to overfitting	DDI prediction, toxicity classification	TWOSIDES, DDIExtraction 2013, FAERS
Deep Learning	DNN, CNN, RNN	Automatic feature extraction, good performance	Requires large data, less interpretable	Drug response prediction, bio-sequence modeling	DeepDDI, DeepPurpose
Graph Neural Networks	GCN, GraphSAGE, GIN	Captures structural information	Training complexity, sensitive to graph quality	Molecular interaction prediction	DDI-KG, DrugRepurposing Hub, Hetionet
Knowledge Graphs	TransE, RotatE, Neo4j-based models	Integrates heterogeneous data, supports reasoning	Costly construction and maintenance	Drug repurposing, interaction inference	Bio2RDF, PharmKG, DrugBank KG

### 4.1 Traditional detection approaches

#### 4.1.1 Clinical trials and pharmacovigilance

Historically, most DDIs have been identified through clinical observation and pharmacovigilance systems. Early-phase clinical trials and preclinical studies typically uncover predictable pharmacokinetic interactions, especially those related to cytochrome P450 (CYP450) isoenzymes. However, these clinical trials often face limitations due to small sample sizes and the exclusion of high-risk patients, such as those with comorbidities or those taking multiple medications, which restricts the generalizability of the findings. Post-marketing surveillance systems, like the FDA’s Adverse Event Reporting System (FAERS), EudraVigilance in the EU, and the WHO’s VigiBase, gather spontaneous reports of adverse drug reactions (ADRs) that may include DDIs. While these platforms play a crucial role in detecting signals of potential interactions, they suffer from issues such as underreporting, selection bias, and challenges in assessing causality. To quantify the strength of these signals, disproportionality analyses—like reporting odds ratios or information components—are frequently employed, but they necessitate additional evidence to establish causal relationships.

#### 4.1.2 *In Vitro* and in vivo experimental models

Experimental pharmacology plays a crucial role in DDIs research. Standard methods include *in vitro* assays that utilize human liver microsomes, recombinant cytochrome P450 (CYP) enzymes, or primary hepatocyte cultures to evaluate enzyme inhibition or induction. Additionally, transporter-based assays that employ cell lines overexpressing P-glycoprotein (P-gp), breast cancer resistance protein (BCRP), organic anion transporting polypeptides (OATP1B1/1B3), or organic cation transporters (OCTs) allow for a detailed understanding of how drugs interact during absorption and excretion (Martins et al., 2022). While *in vivo* animal models offer valuable insights into physiological processes, they are often limited by differences in enzyme expression and transporter distribution across species. As a result, translating findings to human pharmacokinetics frequently requires methods such as allometric scaling or physiologically based pharmacokinetic (PBPK) modeling to ensure accuracy.

### 4.2 Knowledge-based and rule-based systems

CDSS, which are usually integrated with electronic health records (EHRs), depend on carefully curated DDIs knowledge bases to produce alerts for healthcare providers. However, a significant challenge to the effective use of these systems is the absence of a standardized way to represent DDIs data, which hinders interoperability and the ability to reuse this information across different platforms (Hochheiser et al., 2021). These rule-based systems work by comparing prescribed drug pairs with established lists of interactions. Some prominent commercial databases that provide such DDIs information include:

• **Micromedex**–Provides interaction severity, clinical effects, and management strategies.
• **Lexicomp**–Features evidence-graded monographs and clinical recommendations.
• **Stockley’s Drug Interactions**–Offers mechanistic and clinical insight.
• **Drugs. com, Medscape, Epocrates**–Widely used for point-of-care look-up.

Despite their usefulness, rule-based systems often produce a high number of false positives, which can lead to clinician desensitization, commonly referred to as “alert fatigue.” Furthermore, these systems typically do not consider patient-specific factors such as age, organ function, or pharmacogenomics, highlighting the necessity for more flexible and data-driven solutions. CDSS implemented in community pharmacies have demonstrated effectiveness in minimizing adverse drug reactions by offering real-time alerts for potential drug interactions. A scoping review indicates that CDSS tools in these settings can significantly enhance drug safety and mitigate medication-related issues (Moon et al., 2024). Electronic prescription platforms also contribute by delivering immediate feedback on drug interactions, enabling clinicians to make well-informed decisions and avert possible adverse drug interactions. Research has shown that these platforms markedly enhance the safety and effectiveness of clinical decisions (Grammatikopoulou et al., 2024a). Additionally, medication safety initiatives like the EQUIPPED program have proven to significantly decrease adverse drug reactions by broadening the detection of drug interactions across healthcare systems, utilizing both traditional and hub-and-spoke models (Vandenberg et al., 2024).

### 4.3 Computational and machine learning approaches

With the rapid growth of biomedical data, computational models have become essential for predicting DDIs, especially those that have not yet been observed in clinical settings. These models combine various data sources, including chemical structures, biological pathways, electronic health records (EHRs), scientific literature, and gene expression data, to estimate the likelihood of interactions. For instance, the dual graph neural network model developed by Ma and Lei in 2023 effectively integrates molecular structures and drug interaction information, significantly improving the accuracy of DDIs predictions (Ma and Lei, 2023). Similarly, the feature extraction model based on graph neural networks introduced by Al-Rabeah and Lakizadeh in 2022 also enhances the precision of predicting DDIs (Al-Rabeah and Lakizadeh, 2022). To further increase the accuracy and clinical relevance of these predictions, it is crucial to develop more personalized and context-aware models. Muylle et al., in 2021 demonstrated that optimizing the integration of context-specific data can greatly enhance DDIs screening and management through their context-aware clinical decision support system (Muylle et al., 2021). This is further supported by Zhu et al. in 2021, who presented an attribute-supervised tri-factorization model for predicting DDIs (Zhu et al., 2021). Additionally, the AutoDDI model proposed by Gao et al., in 2024, which employs automated graph neural networks, improves both the accuracy and efficiency of DDIs predictions (Gao et al., 2024).

Artificial intelligence (AI) has demonstrated significant promise in the management of polypharmacy, particularly by enhancing the accuracy of predicting drug interactions among elderly individuals and patients with multiple chronic conditions. A review conducted in Saudi Arabia highlighted how AI models can effectively identify high-risk drug combinations associated with polypharmacy (Alsanosi and Padmanabhan, 2024). In this regard, deep learning techniques have made notable strides in predicting DDIs by analyzing complex patterns within extensive datasets. For instance, models like DrugBERT utilize large-scale textual data to achieve a deeper semantic understanding, thereby improving the accuracy of DDIs predictions by extracting relevant information from clinical literature (Liu et al., 2025). Additionally, other models, such as Graph Neural Networks (GNNs), leverage molecular graphs to capture the intricate relationships between different drugs, demonstrating promising results in terms of both accuracy and interpretability (Yao et al., 2024).

The integration of Transformer-based models such as DrugBERT and BioBERT has become increasingly popular, facilitating more advanced interactions and improving predictions through a deeper semantic understanding derived from extensive medical literature (Qi et al., 2025; Hu et al., 2024). These innovations, when paired with efficient natural language processing (NLP) pipelines like DDIExtractor and specialized knowledge bases, enable the early identification of emerging DDIs, often before these interactions are documented in structured clinical or experimental databases.

#### 4.3.1 Similarity-based and network-based approaches

Similarity-based models are founded on the premise that drugs with similar chemical or biological characteristics are more likely to exhibit comparable interactions. To measure drug-drug similarity, various metrics can be derived from molecular fingerprints, profiles of adverse effects, protein targets, and gene ontology annotations. For instance, the KITE-DDI model developed by Zhang et al. (2024) integrates data on chemical structure (Tamir and Yuan, 2024), therapeutic classification, and protein–protein interactions (PPI) within a graph convolutional network (GCN) framework to predict previously uncharacterized DDIs. Furthermore, models that utilize graph neural networks, such as DrugDAGT (Chen et al., 2024), improve the accuracy of drug-target interaction predictions by merging features from drug sequences and three-dimensional structures through a cross-attention mechanism.

Network-based models create interaction graphs, such as those depicting relationships between drugs and targets, enzymes, or phenotypes, and employ algorithms like random walk with restart (RWR), PageRank, or graph embedding techniques like node2vec to uncover new interactions. A notable example is the HDN-DDI model (Sun and Zheng, 2025), which leverages convolutional neural networks and graph neural networks to extract both sequence and three-dimensional structural features of drugs and their targets. By integrating a cross-attention mechanism to combine these multimodal features, the model markedly improves the accuracy of drug-target interaction predictions.

In recent years, several innovative network models have emerged for predicting DDIs. A notable example is the model developed by Tian et al. (2025), which utilizes knowledge graph embedding in conjunction with a convolutional-LSTM network. This approach effectively merges the structural information inherent in knowledge graphs with the temporal characteristics captured by the convolutional-LSTM network. As a result, this model has demonstrated a significant enhancement in the accuracy of multi-label DDIs predictions.

#### 4.3.2 Natural language processing (NLP) and text mining

Natural language processing (NLP) techniques facilitate the automated extraction of DDIs evidence from biomedical literature. Various tools, including DDIExtractor, BioBERT, and SciSpacy, assist in tasks such as named entity recognition (NER), relation extraction, and sentiment analysis across extensive databases like PubMed and ClinicalTrials.gov. The availability of benchmarked datasets, such as the DDIs Extraction 2013 corpus, plays a crucial role in training and evaluating these models. By utilizing NLP-based pipelines, researchers can identify emerging DDIs early on, even before these interactions are added to structured databases.

In recent years, there has been significant progress in the field of DDIs extraction. For instance, Zhang et al. proposed the SCATrans model, which effectively integrates BioBERT, Doc2Vec, and Graph Convolutional Networks (GCN) (Zhang et al., 2025). This model utilizes semantic cross-attention mechanisms to manage multimodal biomedical data, leading to a notable improvement in the accuracy of DDIs predictions. Furthermore, Wang et al. introduced a Transformer-based method for medical entity extraction that combines pre-trained language models with few-shot learning techniques (Wang et al., 2025). This innovative approach has enhanced the capabilities for extracting entities from biomedical literature, showcasing the advancements in this area of research.

#### 4.3.3 Machine learning and deep learning models

Supervised learning models, including support vector machines (SVM), random forests, and gradient boosting, rely on labeled drug-pair data and make use of various features such as molecular descriptors, transcriptomic responses, and side effect profiles for their training processes. Recently, deep learning architectures have shown remarkable benefits in predictive accuracy, particularly when dealing with large-scale and complex datasets. By automating the process of feature learning and effectively modeling intricate nonlinear relationships, deep learning methods have significantly enhanced the accuracy of predicting DDIs.

• **Convolutional Neural Networks (CNNs)**: These methods are utilized to identify spatial or structural hierarchies within drug representations. Convolutional Neural Networks (CNNs) are particularly adept at detecting potential drug interactions by leveraging the structural information inherent in drugs, making them especially useful for analyzing drug images or molecular graphs.
• **Recurrent Neural Networks (RNNs) and Long Short-Term Memory Networks (LSTMs)**: These models are specifically designed to manage sequential data, making them particularly effective for analyzing pharmacokinetic and pharmacodynamic information. Recurrent Neural Networks (RNNs) and Long Short-Term Memory networks (LSTMs) excel at recognizing temporal dependencies, which is crucial when forecasting the long-term effects or interactions of various drugs.
• **Graph Neural Networks (GNNs)**: Graph Neural Networks (GNNs) excel at capturing the topological characteristics present in biomedical interaction graphs. By analyzing the connections between various entities, such as drugs and their targets or enzymes, GNNs can effectively forecast potential interactions between different drugs. This capability allows for a more nuanced understanding of how drugs may influence one another within complex biological systems.
• **Transformer-based Models (e.g., DrugBERT)**: These models effectively capture the semantic relationships found in extensive textual data, including drug leaflets and clinical literature, to identify potential interactions between drugs. This capability makes them particularly adept at extracting intricate drug-drug relationships from medical texts.

Recent hybrid models that integrate graph neural networks (GNNs) with Transformer-based architectures have shown remarkable effectiveness in predicting new and mechanistically plausible DDIs, especially when working with large-scale datasets. These innovative models not only attain high levels of prediction accuracy but also ensure a good degree of interpretability, making them valuable tools in the field of pharmacology.

Despite the excellent predictive capabilities of deep learning models, several challenges remain:

• **Poor Interpretability**: Many deep learning models (e.g., CNNs, RNNs, LSTMs) are “black-box” models, which lack sufficient transparency, making their clinical applicability more challenging.
• **Data Dependency**: Deep learning models heavily rely on large volumes of high-quality data, and their performance can suffer when data is biased or imbalanced.
• **High Computational Resource Requirements**: Training deep learning models typically requires significant computational resources, and the training time can be lengthy, especially when modeling complex drug interactions.

As research in DDIs prediction advances, Deep Learning (DL) methods have emerged as the leading technology in this domain. DL-based DDIs prediction models excel at extracting intricate nonlinear features from large datasets, resulting in high prediction accuracy and robust generalization capabilities. This is especially true when handling diverse data types, including drug molecular structures, gene expression profiles, and pharmacological information, where deep learning models demonstrate considerable strengths. Nevertheless, despite their impressive performance, these models are not without limitations. These limitations are outlined in Table 3, which provides a comprehensive overview of the strengths and weaknesses associated with each model.

**TABLE 3:** The performance indicators of various models in DDI prediction.

Model name	Accuracy	Interpretability	Data type	Application scope	Strengths	Weaknesses
DrugBERT	90%	Low	Text data	Medical literature, EHR	Efficient text analysis, suitable for extracting DDIs from literature	Poor interpretability, relies on large text data
GNN-based Model	85%	Medium	Molecular data, Drug Networks	Drug-target, drug-enzyme networks	High accuracy, captures complex relationships between drugs	Requires large datasets, long training time
CNN-based Model	88%	Medium	Drug structure, Image data	Drug screening and prediction	Good feature extraction capability, suitable for structured data	High computational cost, data structure dependency
RNN/LSTM-based Model	87%	High	Pharmacokinetic data	Drug time-series analysis	Suitable for sequential data, captures dynamic drug behavior	High training complexity, requires sequential data
Hybrid Models (GNN + Transformer)	92%	Medium	Chemical, genomic, clinical data	New drug prediction and interaction analysis	High accuracy, integrates multiple data sources, enhances prediction ability	High model complexity, large computational overhead

### 4.4 Systems pharmacology and knowledge graphs

Systems pharmacology aims to model the effects of drugs within interconnected biological networks by integrating pharmacokinetics (PK) and pharmacodynamics (PD). Knowledge graphs (KGs) serve as a semantic representation of the relationships among drugs, targets, pathways, diseases, and phenotypes. To effectively encode these semantic relationships and facilitate cross-platform integration, standardized information models are crucial (Hochheiser et al., 2021). Although KGs enhance reasoning capabilities and interpretability, their construction and maintenance demand significant expert curation and computational resources. Notable platforms that support these efforts include:

• **DrugBank and PharmGKB**–Curated molecular and clinical pharmacology data.
• **Hetionet and Bio2RDF**–Multiscale biological integration.
• **DDI-KG**–A specialized KG for DDIs prediction via link prediction models (e.g., TransE, RotatE, ComplEx).

These tools facilitate explainable and mechanistically grounded inference of DDIs. The SAO semantic structure-based forecasting method offers a robust framework for predicting adverse drug reactions, especially in cases of polypharmacy, where the risks associated with drug interactions can be intricate and challenging to detect (Wang JY. et al., 2024).

In recent years, AI-based knowledge graph mining methods have demonstrated significant promise in predicting DDIs. By utilizing deep learning techniques and graph neural networks (GNNs), researchers can automatically analyze extensive biomedical data to identify relationships between various drugs, thereby aiding in the discovery of potential DDIs. Some of the prevalent AI-based methods include:

• **Graph Neural Networks (GNNs)**: GNNs excel at identifying intricate, nonlinear relationships among entities, including drugs, targets, and diseases within knowledge graphs. By utilizing node and edge embeddings, GNNs can effectively forecast various types of DDIs. For example, methods such as GraphSAGE and Graph Convolutional Networks (GCN) are commonly employed for the classification and prediction of DDIs, demonstrating their utility in this domain (Wang Yaqing et al., 2024).
• **Natural Language Processing (NLP) Combined with Knowledge Graphs**: Integrating natural language processing (NLP) techniques, particularly those utilizing the Transformer architecture like BERT and GPT, enables the extraction of potential drug interactions from biomedical literature. The combination of NLP with knowledge graphs (KGs) facilitates the automatic identification of intricate relationships between drugs, thereby revealing interactions that may have been previously overlooked (Xu et al., 2024; Abdullahi et al., 2025; Wan et al., 2024; Zhou et al., 2024).
• **Reinforcement Learning**: Reinforcement learning methods are employed to simulate various scenarios of DDIs, adjusting model parameters according to the predicted outcomes. This technique proves valuable in uncovering unknown interactions within dynamic drug interaction networks. Its application in drug discovery and drug repurposing has yielded new insights into the prediction of complex drug reactions (Inoue et al., 2025; Zhu F. et al., 2024).

These AI methods improve the accuracy of drug-drug interaction (DDI) predictions and offer a scientific foundation for personalized medication therapy, especially in situations involving multiple medications.

### 4.5 Real-world data and EHR mining

Electronic health records provide valuable real-world evidence that can help identify potential DDIs. By retrospectively analyzing medication histories, lab results, and clinical outcomes, researchers can uncover associations that may not have been recognized before. Time-aware models, particularly those based on transformer architectures, are capable of capturing the temporal aspects of drug exposure and the associated risks of interactions. Nonetheless, there are challenges to consider, such as the variability in data, missing information, and confounding factors. To address these issues, methods like federated learning and differential privacy are being investigated to facilitate large-scale DDI analysis while preserving patient privacy across different institutions. Additionally, electronic prescription systems are crucial for monitoring DDIs, especially through real-time alerts that notify healthcare providers of potential risks. A study conducted in Greece demonstrated that CDSS can significantly enhance the accuracy of clinical decision-making, particularly in managing DDIs (Grammatikopoulou et al., 2024b).

### 4.6 Integration of pharmacogenomics and personalized DDI risk

Pharmacogenomic (PGx) variability is essential in understanding how individuals respond differently to DDIs. Variations in genes like CYP2C9, CYP2D6, and SLCO1B1 can significantly influence how drugs are metabolized or transported in the body. For example, individuals who are poor metabolizers of CYP2D6 may face a higher risk of experiencing toxicity from medications such as codeine or tricyclic antidepressants. By incorporating PGx data into clinical DDIs risk models, healthcare providers can enhance precision medicine, tailoring treatments to individual genetic profiles. Tools like PharmCAT and CPIC guidelines support the integration of genetic information into treatment decisions, which is particularly important in high-risk areas such as oncology, psychiatry, and cardiology. Personalized treatment strategies are vital for managing complex drug interactions, especially among psychiatric patients. Research by Cuomo et al. (2024) highlights that clinical responses to vortioxetine can vary widely among different patient profiles. In psychiatric patients with additional health conditions, the risk of DDIs may increase due to the simultaneous use of antidepressants and medications for physical illnesses (Berk et al., 2023). Furthermore, the growing use of psychedelics, whether prescribed or recreational, underscores the need for a better understanding of potential DDIs and effective communication between clinicians and patients (Boehnke et al., 2023). Additionally, gender-diverse individuals undergoing hormone therapy may present unique DDIs profiles, particularly in the context of psychiatric pharmacotherapy (Kim et al., 2023).

### 4.7 Challenges and future directions

Despite substantial progress, several challenges remain:

• **Data quality and standardization**–Heterogeneous ontologies and inconsistent annotations hinder model interoperability. Efforts toward minimal information models for DDIs are a step toward solving these limitations (Hochheiser et al., 2021).
• **Model interpretability**–Black-box algorithms reduce clinician trust and limit regulatory acceptance.
• **Rare and population-specific DDIs**–Existing models often fail to detect infrequent or demographically restricted interactions.
• **Experimental validation**–In silico predictions require rigorous *in vitro*, *in vivo*, or real-world corroboration.

Looking ahead, the integration of artificial intelligence with systems pharmacology, digital twin modeling, and real-time electronic health record (EHR) analytics presents promising opportunities for the advancement of next-generation DDIs surveillance. Key elements such as explainable AI (XAI), causal inference frameworks, and collaboration with regulatory bodies will be essential in maintaining clinical relevance, ensuring transparency, and prioritizing patient safety. Recent advancements in artificial intelligence, particularly in areas like graph neural networks and multimodal deep learning, have notably enhanced the accuracy of predicting potential drug-drug interactions (Zhang et al., 2023; Wang N-N. et al., 2024; Huang et al., 2023; Zhao et al., 2024; Li et al., 2023). Additionally, insights from patients and caregivers play a vital role in recognizing and addressing DDI-related risks, especially in outpatient settings (Sharma et al., 2024).

As biomedical data continues to expand, the challenge of analyzing health data across different institutions while protecting data privacy has become increasingly critical. Traditional methods of data analysis often involve centralized data storage, which poses significant privacy risks. In contrast, federated learning techniques enable data to be processed locally, thereby preserving patient privacy and facilitating data sharing between institutions. This approach is especially vital for predicting and managing drug-drug interactions. For instance, the privacy-preserving federated learning framework introduced by Sinaci et al. (2024) effectively tackles privacy concerns associated with cross-institutional data collaboration, all while improving the efficiency of data analysis.

## 5 Special populations and context-specific considerations in DDI research

DDIs pose a significant and evolving challenge in clinical pharmacology, especially due to the diverse nature of patient populations. Certain groups, such as the elderly, pediatric patients, pregnant and lactating women, individuals with liver or kidney dysfunction, and those with genetic variations affecting drug metabolism, often show differences in how their bodies process medications (pharmacokinetics) and respond to them (pharmacodynamics) (Strawn et al., 2021). These differences can greatly affect both the likelihood of experiencing DDIs and their clinical consequences. Additionally, specific clinical situations, such as the use of multiple medications in cancer treatment, critical care settings, and variations in treatment practices across different regions, add to the complexity of managing DDIs. Therefore, a detailed understanding of these population-specific and contextual factors is essential for improving personalized medication therapy and ensuring that regulatory practices are based on solid evidence.

**FIGURE 5:**
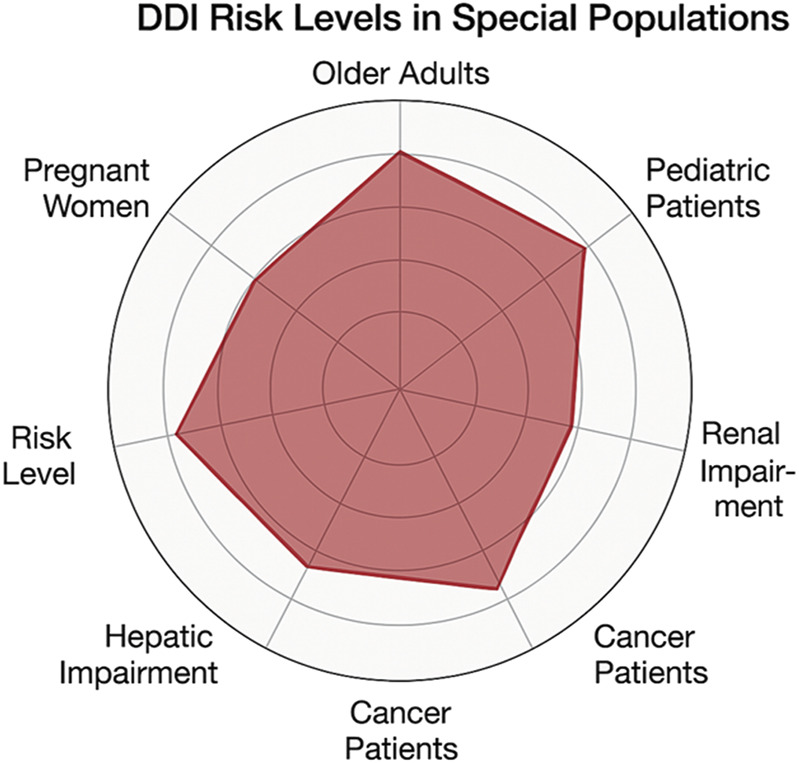
Radar chart illustrating relative risk levels of DDIs in special populations. Risk scores range from 1 (low) to 5 (very high) and are derived from published literature and clinical evidence.

To illustrate the varying susceptibility of different populations to clinically significant DDIs, we created a comparative radar chart (Figure 5). This chart combines factors such as physiological changes, the prevalence of polypharmacy, and risk estimates derived from existing literature. Our findings indicate that the elderly and patients experiencing polypharmacy are at the greatest risk, followed closely by individuals in oncology and those with organ impairments.

### 5.1 Elderly patients: polypharmacy and physiological alterations

#### 5.1.1 Pharmacokinetic and pharmacodynamic changes in aging

Physiological aging brings about significant changes in the body that can affect how drugs are processed and increase the risks of DDIs. For example, as people age, there is a decrease in liver blood flow, kidney function, and alterations in body composition, all of which can influence drug metabolism and clearance. A notable consequence of aging is the decline in glomerular filtration rate, which can hinder the elimination of drugs that are primarily excreted by the kidneys, like digoxin. This impairment raises the potential for toxicity, especially when these drugs are taken alongside other medications that may interact with them. Additionally, aging can lead to changes in how receptors respond to drugs and can disrupt the body’s ability to maintain balance, particularly affecting drugs that act on the central nervous system (CNS) (Zerah et al., 2021). This is especially relevant for medications such as benzodiazepines, opioids, and antipsychotics, where the altered sensitivity and homeostatic responses can heighten the risk of adverse effects and interactions.

#### 5.1.2 Polypharmacy and inappropriate prescriptions

Polypharmacy, which is commonly defined as the simultaneous use of five or more medications, is particularly common among older adults and significantly increases the risk of DDIs. This issue is especially pronounced in elderly patients. Research by Abdu et al. highlights that polypharmacy is a major risk factor for DDIs in older populations (Abdu et al., 2025). For example, a cross-sectional study conducted in the U.S. found that 62.7% of elderly patients with cardiovascular disease were subjected to polypharmacy, with 34.8% experiencing at least one severe potential drug-drug interaction. To help identify potentially inappropriate medications (PIMs), clinical tools like the Beers Criteria and the STOPP/START guidelines are utilized, as many of these medications are known to cause significant interactions. A specific example is the combination of warfarin with trimethoprim-sulfamethoxazole, which can lead to increased bleeding due to CYP2C9 inhibition and changes in gut microbiota. Similarly, taking citalopram alongside omeprazole may heighten the risk of QT interval prolongation, particularly in patients with diminished CYP2C19 activity. The most frequently encountered potential drug-drug interactions involve warfarin being co-prescribed with other interacting agents such as nonsteroidal anti-inflammatory drugs (NSAIDs) or antibiotics, highlighting the critical need for structured medication reviews in geriatric care (Sheikh-Taha and Asmar, 2021). A European multicenter study found that 54.8% of elderly patients had at least one potentially clinically significant drug-drug interaction before being admitted to the hospital, and this figure rose to 58.3% during their stay. Antithrombotic agents were involved in 40.6% of these significant interactions, particularly when they were taken alongside non-steroidal anti-inflammatory drugs (NSAIDs) or antibiotics. The use of STOPP/START criteria in the OPERAM trial highlighted their effectiveness in pinpointing potentially inappropriate medications and related drug-drug interactions. While multidose drug dispensing systems are designed to minimize errors associated with polypharmacy, they may not sufficiently warn users about the risks of drug-drug interactions (Martin-Oliveros et al., 2024). Furthermore, there is a strong link between polypharmacy and the use of potentially inappropriate medications (PIMs) and an increased risk of falls among older adults (AlHarkan et al., 2023).

### 5.2 Pediatric population: developmental pharmacology

#### 5.2.1 Maturation of drug metabolism and transport

Children, especially neonates and infants, experience significant developmental changes in their drug-metabolizing enzymes and transporters. For instance, enzymes like CYP3A7, CYP2D6, and CYP1A2 show variations in their expression levels depending on the child’s age, which can lead to differences in how susceptible they are to DDIs. Additionally, the underdevelopment of renal transport mechanisms, such as organic anion transporters (OATs), organic cation transporters (OCTs), and multidrug and toxin extrusion proteins (MATEs), affects how drugs are cleared from the body. A notable example of this is the increased risk of ototoxicity when aminoglycosides are used in conjunction with loop diuretics in neonates. This risk arises from both the immature excretory functions of their kidneys and the synergistic nephrotoxic effects of these medications.

#### 5.2.2 Off-label drug use and limited DDI data

Off-label drug use is prevalent in pediatrics, primarily because children are often excluded from numerous clinical trials, leading to a scarcity of pediatric-specific data on DDIs. This lack of information compels healthcare providers to rely on data derived from adult populations, which can be unsuitable due to the developmental differences between children and adults. Although legislative measures like the Pediatric Research Equity Act (PREA) and Pediatric Investigation Plans (PIPs) have made strides in tackling this challenge, significant gaps in knowledge and data still persist.

### 5.3 Pregnant and lactating women: dual-physiology and teratogenic risk

#### 5.3.1 Pregnancy-induced physiological changes

Pregnancy brings about significant changes in the body that can affect how drugs are processed. For instance, during pregnancy, the activity of the enzyme CYP3A4 increases while that of CYP1A2 decreases. Additionally, there is an increase in renal blood flow and plasma volume, all of which can alter how drugs are metabolized and how they interact with one another. A practical example of this is that the heightened activity of CYP3A4 can lead to lower levels of midazolam, which in turn can change how this drug interacts with inhibitors of CYP3A4. Moreover, DDIs involving teratogenic medications, such as valproate and isotretinoin, along with enzyme inhibitors, can increase the exposure of the fetus to these drugs, highlighting the importance of careful risk-benefit evaluations. Furthermore, interactions that affect placental transporters, such as BCRP and P-glycoprotein (P-gp), can also influence the amount of medication that reaches the fetus.

#### 5.3.2 Lactation and breastmilk transfer

Drugs can transfer into breast milk through two primary mechanisms: passive diffusion and transporter-mediated processes. DDIs that raise maternal drug concentrations or modify the composition of breast milk can significantly affect the amount of medication that a newborn is exposed to. A notable example of this is the interaction between fluoxetine, an antidepressant, and metoclopramide, a medication often used to treat nausea. This interaction may lead to an increase in prolactin secretion, which could have implications for lactation and the amount of the drug that an infant ingests through breast milk.

### 5.4 Patients with hepatic or renal impairment

#### 5.4.1 Hepatic impairment and reduced metabolic capacity

Liver dysfunction affects both phase I and phase II metabolic pathways, leading to significant implications for drug metabolism. Enzymes like CYP1A2 and CYP2C19 are especially vulnerable to the suppressive effects of cirrhosis, which can result in unpredictable and potentially dangerous DDIs when these enzymes are induced or inhibited. This is particularly concerning for medications that have narrow therapeutic windows, such as carbamazepine, phenytoin, and propranolol, as they require meticulous monitoring in patients with liver impairment to avoid adverse effects and ensure therapeutic efficacy.

Recent advances in transcriptomic profiling have led to the creation of tools like the TGx-DDI biomarker, which is capable of characterizing drug-induced DNA damage responses in human HepaRG™ liver cells. This biomarker may provide valuable insights into the risks associated with DDIs in conditions where liver function is compromised (Buick et al., 2021).

#### 5.4.2 Renal dysfunction and drug accumulation

Chronic kidney disease (CKD) leads to a decrease in drug clearance and changes in protein binding, which increases the likelihood of drug accumulation and toxicity. The use of nephrotoxic medications, such as aminoglycosides, nonsteroidal anti-inflammatory drugs (NSAIDs), and contrast agents, can further worsen kidney damage. Additionally, the presence of uremia can hinder the function of transporters and enzymes, making it essential to implement individualized therapeutic drug monitoring (TDM) that considers the potential for DDIs.

### 5.5 Oncology and immunocompromised populations

#### 5.5.1 Anticancer polypharmacy and enzyme modulation

Oncology patients often undergo complex treatment regimens that include chemotherapy, targeted therapies, antimicrobials, and supportive care agents. A significant number of anticancer drugs, particularly tyrosine kinase inhibitors (TKIs), are either metabolized by or influence the activity of CYP3A4, which makes them susceptible to serious DDIs with medications such as azoles or macrolides. Additionally, immunosuppressants like tacrolimus and cyclosporine have narrow therapeutic indices and can interact adversely with antifungals, calcium channel blockers, or antiepileptics. Research indicates that over 60% of psychiatric inpatients encounter at least one clinically significant potential drug-drug interaction, frequently involving psychotropic medications like antipsychotics, selective serotonin reuptake inhibitors (SSRIs), and benzodiazepines, highlighting the necessity for integrated decision-support tools in psychiatric care. In the field of oncology, polypharmacy is prevalent, and employing comprehensive care strategies, such as CDSS, can help reduce the risks associated with drug-drug interactions. A qualitative study conducted in Hong Kong shed light on the challenges and strategies for enhancing drug safety in outpatient oncology care (Ho et al., 2024). In treating bipolar depression, drug interactions can significantly affect treatment outcomes. A drug surveillance project in Bavaria revealed that patients undergoing polypharmacy for bipolar depression face a heightened risk of adverse drug interactions, especially with antidepressants (Kriner et al., 2024). Furthermore, in managing multiple sclerosis, patient preferences for therapies, including sphingosine-1-phosphate receptor modulators, are often shaped by the potential for drug interactions, particularly when these therapies are used alongside other medications for comorbid conditions (Keenan et al., 2024). For example, serotonin syndrome can occur due to pharmacodynamic interactions between SSRIs and other serotonergic drugs (Zhu A. et al., 2024).

#### 5.5.2 Drug–microbiome interactions

The gut microbiome plays a crucial role in drug metabolism, and its disruption through antibiotics or chemotherapy can significantly affect drug deconjugation and enterohepatic circulation. For instance, bacteria that produce β-glucuronidase can increase the toxicity of irinotecan, highlighting a new category of microbiome-mediated DDIs in oncology. In addition, the use of traditional medicine is prevalent among diabetes patients, particularly in Africa, which can lead to interactions with conventional medications. Unfortunately, these interactions are often overlooked, resulting in adverse drug reactions and complications in treatment (Ekpor et al., 2024). The significance of gut microbiota in drug metabolism and interactions is gaining recognition. Probiotics have been found to modify drug efficacy and toxicity, and research conducted in Serbia indicates that healthcare students are aware of these interactions (Danic et al., 2024); however, there is a need for further education on the subject. Knowledge regarding the interactions between drugs and dietary supplements is still insufficient among healthcare professionals, which can lead to an increased risk of adverse reactions due to overlooked interactions between supplements and prescription medications. Studies reveal that many healthcare workers are not fully aware of these risks (Büyükkasap and Yazici, 2024). Herbal products further complicate DDIs, particularly in psychiatric or immunocompromised patients, where modulation of cytochrome enzymes can disrupt standard therapies (Patel et al., 2024). Cannabinoid compounds, such as THC and CBD, have the potential to inhibit CYP enzymes, increasing the likelihood of metabolic DDIs (Smith and Gruber, 2023). Patients with HIV often navigate complex antiretroviral therapy (ART) regimens, where even minor errors related to DDIs can significantly impact therapeutic efficacy (Chastain et al., 2024). Moreover, the widespread use of dietary supplements among older adults presents substantial, yet often unrecognized, risks for DDIs (Fravel et al., 2023).

### 5.6 Intensive care and emergency settings

#### 5.6.1 Complex regimens and organ support devices

Critically ill patients often receive a variety of intravenous medications, which can lead to both pharmacokinetic and physicochemical interactions. The use of medical devices like renal replacement therapy (RRT), extracorporeal membrane oxygenation (ECMO), and plasma exchange can significantly change how drugs are cleared from the body, making it essential to adjust dosages and assess potential DDIs carefully. Emergency department physicians who follow national guidelines for HIV post-exposure prophylaxis can notably lower the chances of adverse drug interactions. Research indicates that adhering to these guidelines not only enhances patient safety but also improves overall outcomes by reducing the likelihood of harmful drug interactions (Heck et al., 2024). Furthermore, pharmacist-led stewardship programs are becoming increasingly important in managing and minimizing DDIs related to antiretroviral therapy (ART) (Ahmed et al., 2023).

#### 5.6.2 Time-critical decision-making

The urgent nature of emergency care often limits the ability to conduct comprehensive evaluations of DDIs. To address the risks associated with these interactions in high-pressure environments, it is crucial to implement advanced CDSS that include severity stratification and tailored patient alerts. These systems can help healthcare providers quickly identify and manage potential interactions, ensuring safer and more effective patient care during emergencies.

### 5.7 Pharmacogenomic subpopulations

Polymorphic variants in drug-metabolizing enzymes, such as CYP2C9/2C19, CYP2D6, and CYP3A5*3, play a crucial role in influencing the risk of drug-drug interactions (DDIs). For instance, individuals identified as poor metabolizers of CYP2D6 may be at an increased risk of developing serotonin syndrome when paroxetine is used in conjunction with tramadol. By incorporating pharmacogenomic (PGx) profiling into standard healthcare practices, utilizing platforms like PharmCAT or YouScript, healthcare providers can conduct personalized risk assessments and make proactive dose adjustments tailored to individual patient needs.

### 5.8 Global considerations and population diversity

#### 5.8.1 Ethnopharmacology and DDI sensitivity

Genetic variations in metabolic enzymes, along with dietary habits and the common use of traditional medicines, play significant roles in the differences in DDIs risks among various ethnic groups. For example, individuals from East Asian backgrounds often possess a higher frequency of poor metabolizer genotypes for the CYP2C19 enzyme, which can diminish the effectiveness of clopidogrel when it is taken alongside proton pump inhibitors. Additionally, herbal remedies like St. John’s Wort and ginseng can either induce or inhibit cytochrome P450 enzymes, making the management of DDIs even more challenging. Beyond these genetic and cultural factors, differences in enzyme expression and drug transporter activity based on sex and gender can also influence DDI risks. For instance, the levels of estrogen and testosterone can impact the activity of CYP enzymes, which is particularly relevant for both cisgender and transgender patients undergoing hormone therapy (Cirrincione and Huang, 2021).

#### 5.8.2 Resource-limited settings

In low- and middle-income countries (LMICs), the challenges of limited access to DDIs screening tools, combined with the co-treatment of infectious and chronic diseases, heighten the risks associated with polypharmacy. This issue is especially critical for older adults, who frequently face a higher prevalence of DDIs due to the presence of multiple health conditions and instances of inappropriate prescribing practices. Recently, an international expert panel has put together a consensus list of potentially clinically significant drug-drug interactions specifically for the elderly. This list provides a standardized framework that could be used as a basis for developing simplified DDI screening tools tailored for resource-limited environments (Anrys et al., 2021). By integrating these curated interaction lists into mobile applications that are adapted for different languages, we could take a significant step toward enhancing medication safety in LMICs.

### 5.9 Future directions in personalized DDI management

• **Dynamic DDIs Modeling**: Integration of real-time PK/PD data and digital phenotyping for adaptive therapy.
• **Wearable and Sensor Technologies**: Early detection of DDIs effects, such as QT prolongation.
• **Global DDIs Surveillance Networks**: Real-world evidence from diverse populations to improve alert accuracy.
• **Patient-Centric Tools**: Mobile DDIs checkers and at-home genetic screening to empower informed decision-making.

## 6 Regulatory considerations and DDI labeling strategies

The accurate identification and proactive management of clinically significant DDIs is a crucial priority for health authorities worldwide. Regulatory frameworks have evolved to facilitate the systematic evaluation of DDIs at every stage of drug development, from preclinical studies to post-marketing surveillance. This section outlines the main strategies employed by prominent regulatory agencies, including the FDA, EMA, and PMDA, while also noting regional differences and the trend towards global harmonization. It is important to mention that this content is not recommended for citation unless it is connected to socioeconomic factors in drug interaction management (Leslie, 2024). Efforts to harmonize ontological frameworks, such as RxNorm and DrugBank, play a key role in maintaining consistency in DDIs knowledge bases (Kawakami et al., 2024). Additionally, this section discusses the growing role of artificial intelligence (AI) in predicting DDIs and the regulatory considerations that come with its implementation.

### 6.1 United States: FDA guidance

The U.S. Food and Drug Administration (FDA) has established a thorough and risk-informed framework for evaluating drug-drug interactions (DDIs), which includes both laboratory (*in vitro*) and clinical (*in vivo*) studies. The guidance updated in 2020 highlights the importance of using physiologically based pharmacokinetic (PBPK) modeling as a key method for predicting interactions that occur due to metabolism and transport processes. The FDA supports model-informed drug development (MIDD), which combines PBPK with quantitative systems pharmacology (QSP) to enhance the design of studies and improve regulatory decision-making. Additionally, the FDA has promoted the use of artificial intelligence (AI)-driven models that utilize extensive clinical data to facilitate real-time detection of DDIs.

The FDA is progressively incorporating AI tools into the assessment of DDIs, especially within CDSS. To ensure these AI models are effective in real-world settings, they must undergo thorough validation that includes not just data from clinical trials but also real-world evidence (RWE) sourced from electronic health records (EHRs). This approach helps confirm that the AI tools are applicable in everyday clinical practice. Additionally, the FDA emphasizes the necessity for clear labeling that accurately conveys the underlying mechanisms of the identified DDIs and their therapeutic consequences. It is crucial that AI-generated predictions are presented in a way that is clinically actionable, thereby providing valuable support to both healthcare providers and patients.

### 6.2 European Union: EMA framework

The European Medicines Agency (EMA) offers comprehensive guidance on evaluating pharmacokinetic interactions, placing significant importance on mechanistic classification, especially concerning cytochrome P450 enzymes and drug transporters. Within the EMA framework, there is a clear requirement for strong justification when waiving clinical interaction studies, frequently necessitating sensitivity analyses or supplementary modeling to substantiate these claims. In contrast to the FDA, the EMA applies more stringent regulatory oversight regarding the reasoning behind the decision not to perform certain clinical trials.

The European Medicines Agency (EMA) has begun to integrate artificial intelligence (AI) into the evaluation of DDIs, acknowledging its ability to predict intricate interactions and categorize DDIs based on underlying mechanisms. Nevertheless, the application of AI tools must be firmly rooted in thorough scientific validation and must adhere to the agency’s commitment to transparency and data sharing. To support this initiative, the EMA provides an open-access DDIs database, which aids in the incorporation of AI-driven tools designed to uncover previously underreported or newly emerging DDIs.

### 6.3 Japan: PMDA requirements

The Pharmaceuticals and Medical Devices Agency (PMDA) in Japan generally aligns with international best practices while also taking into account specific regional considerations. A key focus for the PMDA is ethnic sensitivity; they often require bridging studies to ensure that DDIs data can be accurately extrapolated to Japanese populations. Additionally, the PMDA has been increasingly supportive of modeling and simulation techniques, including the use of AI-based systems, to predict potential DDIs effectively.

The PMDA has approached the endorsement of AI-driven models with caution, yet it is gradually advancing towards supporting these technologies. These models have the potential to incorporate population-specific data, which is particularly important for enhancing DDIs predictions. This is especially relevant in the context of Japanese populations, where unique genetic factors can significantly influence drug metabolism.

### 6.4 Comparison and global harmonization trends

While national regulatory agencies have their own specific nuances, there is a growing trend towards convergence in DDIs evaluation strategies, largely influenced by ongoing efforts to harmonize guidelines through the International Council for Harmonisation (ICH). The upcoming ICH M12 guideline marks a significant step forward, as it seeks to standardize the scientific principles that govern DDIs study design, data interpretation, and labeling requirements. Among the leading regulatory bodies, the U.S. Food and Drug Administration (FDA) has taken a proactive approach by advocating for model-informed and simulation-based regulatory science. In contrast, the European Medicines Agency (EMA) and the Pharmaceuticals and Medical Devices Agency (PMDA) are gradually incorporating these methodologies, reflecting their unique institutional and regional priorities. Despite these differences, it is crucial for these agencies to align their efforts to reduce redundant testing, speed up global drug development, and maintain consistency in communicating clinical risks. As these global agencies strive for harmonization, recent regulatory guidance—such as the FDA’s protocols for CYP450 and transporter studies (U.S. Food and Drug Administration FDA, 2020)—illustrates a developing consensus. Additionally, emerging artificial intelligence tools, like the affinity models summarized on arXiv, provide further translational benefits in this evolving landscape (Vefghi et al., 2025).

There is a growing movement aimed at achieving global consistency in the use of artificial intelligence (AI) tools for predicting DDIs. AI-driven models must comply with international regulations concerning data privacy, transparency of the models, and their validation processes. New AI technologies, such as graph neural networks (GNNs) and transformer-based models, provide significant advantages, allowing for more precise and context-sensitive predictions of DDIs. As international organizations strive for harmonization, it is essential that these AI models adhere to the same stringent standards that are applied to conventional DDIs assessment tools, thereby ensuring their relevance in clinical settings and safeguarding patient safety.

## 7 Limitations and future directions

Despite significant advancements in DDIs research, several key limitations persist that hinder clinical implementation, regulatory harmonization, and the relevance of findings in real-world settings.

### 7.1 Limitations

Many current DDIs prediction models are based on limited and skewed datasets, which primarily focus on well-characterized drug classes. This focus leads to a significant underrepresentation of rare interactions, population-specific pharmacokinetics, and the complexities of real-world treatment scenarios. Additionally, clinical trials frequently exclude vulnerable populations, including the elderly, pediatric patients, pregnant individuals, and those with multiple health conditions. Such exclusions undermine the external validity of DDIs evidence, particularly for high-risk groups.

A considerable number of machine learning-based DDIs prediction models face challenges related to interpretability, which diminishes trust among clinicians and hampers acceptance by regulatory bodies. These models frequently function as “black boxes,” delivering precise predictions without offering insights into their underlying mechanisms or pharmacological reasoning. Consequently, this lack of transparency restricts their incorporation into clinical workflows and the labeling of drugs.

Current DDIs frameworks inadequately address the role of herbal medicines, dietary supplements, and traditional therapies, even though these are commonly used, especially in low- and middle-income countries (LMICs). The diversity in therapeutic practices across different geographic and cultural contexts adds another layer of complexity that is seldom reflected in conventional pharmacovigilance systems or regulatory guidelines.

Most DDIs risk stratification tools are static, which means they do not adapt to the dynamic physiological changes, evolving comorbidities, or treatment adjustments that patients experience over time. Currently, the integration of real-time patient data, including electronic health records (EHRs), therapeutic drug monitoring (TDM), and longitudinal health trends, remains underutilized in modern DDIs management systems. This lack of adaptability and real-time data incorporation limits the effectiveness of these tools in providing personalized and timely assessments of DDIs risks, ultimately impacting patient safety and treatment outcomes.

### 7.2 Future directions

Future research should concentrate on creating explainable and clinically validated DDIs prediction models that combine mechanistic pharmacology with sophisticated data-driven techniques. It is essential to prioritize the generalizability of these models across various patient populations and clinical contexts, including intensive care units, oncology settings, and resource-limited environments.

The convergence of pharmacogenomics, mobile health technologies, and digital infrastructure presents remarkable opportunities for personalized assessment of DDIs risks. By integrating genotype-guided dosing algorithms with real-time decision support tools and mobile patient engagement platforms, we can facilitate a dynamic and individualized approach to pharmacovigilance. This integration allows healthcare providers to tailor medication plans based on a patient’s genetic profile, ensuring safer and more effective treatment options while actively engaging patients in their healthcare journey.

The establishment of global DDIs surveillance networks, along with the implementation of interoperable data standards, can significantly improve consistency across different regulatory jurisdictions. It is essential for regulators, clinicians, informaticians, and technology developers to work together across sectors to effectively translate computational advancements into practical tools that enhance the safety of pharmacotherapy.

**FIGURE 6:**
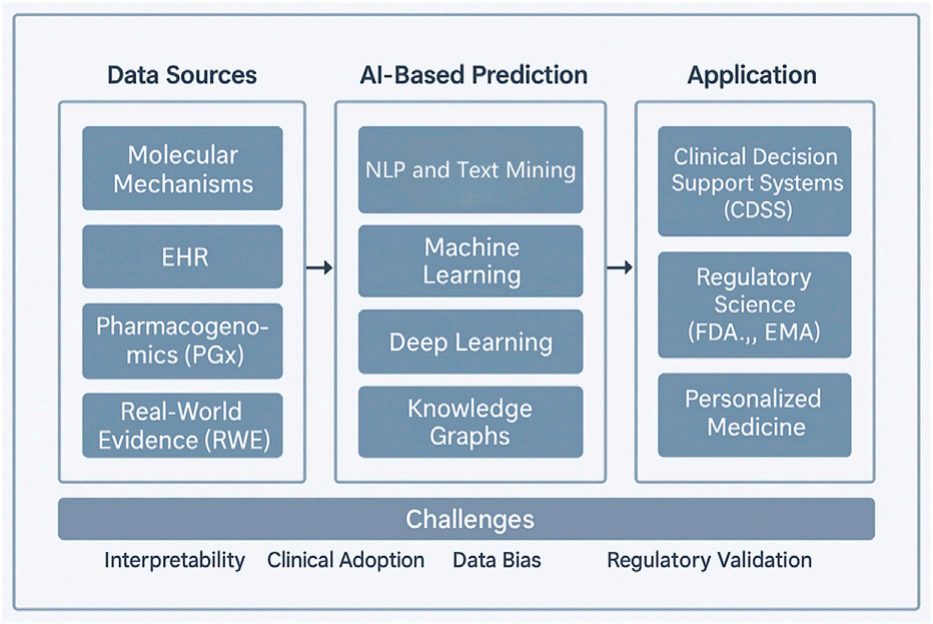
A proposed integrative framework for drug–drug interaction (DDI) prediction and clinical translation. An integrative framework for drug–drug interaction (DDI) prediction and clinical translation. The framework is structured in three tiers: (1) Data Inputs, including molecular mechanisms, electronic health records (EHR), pharmacogenomics (PGx), and real-world evidence (RWE), which serve as foundational sources for model development; (2) Predictive Modeling, encompassing rule-based systems, machine learning, deep learning, and knowledge graphs, enabling scalable and mechanistically informed DDI predictions; and (3) Application Domains, where model outputs inform clinical decision support systems (CDSS), regulatory science, and personalized medicine. Cross-cutting challenges—such as interpretability, clinical adoption, data bias, and regulatory validation—remain critical to successful implementation. This framework supports a dynamic, data-driven pathway from mechanistic understanding to patient-specific pharmacovigilance.

To tackle the aforementioned challenges, we propose an integrative translational framework that connects diverse data sources, predictive modeling strategies, and various application domains. This framework, illustrated in Figure 6, is designed to enhance the prediction of DDIs through the use of AI-powered models. It effectively integrates heterogeneous data sources with AI-based prediction methodologies and real-world applications, emphasizing the challenges faced and the solutions available for translating these insights into clinical practice.

### 7.3 Data sources

The foundational data inputs essential for DDIs prediction come from various sources:

• **Molecular Mechanisms**: The data encompasses how drugs interact at the molecular level, including details about chemical structures, biological pathways, and the interactions that occur within biological systems.
• **Electronic Health Records (EHRs)**: Patient data, including medical history, laboratory results, and prescription records, are crucial for understanding drug interactions in diverse patient populations.
• **Pharmacogenomics (PGx)**: Genetic information that influences drug metabolism, efficacy, and toxicity. This data helps predict individual responses to drugs.
• **Real-World Evidence (RWE)**: Data derived from actual clinical practice, including observational studies and post-market surveillance, to capture drug interactions in broader patient populations.

### 7.4 AI-based prediction

The second section of the framework involves **AI-based prediction methods** that process the heterogeneous data sources mentioned above. These methods include:

• **Machine Learning**: Algorithms that learn from large datasets to predict drug interactions based on patterns and correlations.
• **Deep Learning**: A subset of machine learning that uses neural networks to model complex, high-dimensional data and improve the prediction of interactions.
• **Knowledge Graphs**: Graph-based models that represent the relationships between drugs, diseases, genes, and other medical entities, providing a structured approach to explore interactions.
• **Natural Language Processing (NLP) and Text Mining**: These methods are used to extract valuable insights from unstructured data such as scientific literature, clinical notes, and drug labels.

### 7.5 Applications

The predictions generated by AI-based models are directed toward practical applications:

• **CDSS**: AI-powered tools that assist healthcare providers in making informed decisions about drug prescriptions, minimizing harmful interactions.
• **Regulatory Science (FDA, EMA)**: Regulatory bodies can use AI models to enhance drug approval processes, ensuring safety and efficacy based on predicted interactions.
• **Personalized Medicine**: AI predictions can be applied to tailor drug regimens for individual patients, considering their genetic background, medical history, and potential for drug interactions.

### 7.6 Challenges

While the framework holds promise, several challenges remain in implementing AI-powered DDI predictions:

• **Interpretability**: AI models, especially deep learning, can be difficult to interpret, making it challenging to understand the rationale behind predictions.
• **Clinical Adoption**: Integrating AI-based prediction tools into clinical workflows requires overcoming barriers such as clinician trust, training, and workflow integration.
• **Data Bias**: Models trained on biased data may perpetuate existing healthcare disparities, leading to inaccurate predictions for certain patient populations.
• **Regulatory Validation**: AI-driven predictions need rigorous validation to meet regulatory standards for clinical applications, ensuring their safety and efficacy in real-world use.

## 8 Emerging trends and future perspectives in DDI research

As pharmacotherapy becomes increasingly complex due to the rise of polypharmacy, biologics, and personalized medicine, traditional paradigms for understanding DDIs are encountering significant limitations. In response to these challenges, several emerging trends are transforming DDIs research into more integrative, predictive, and patient-centered approaches. For instance, insights from pharmacogenetics regarding atypical antipsychotics can enhance our ability to predict DDIs risks and tailor dosing to individual patients, thereby improving treatment outcomes and minimizing adverse effects (Vasiliu, 2023).

### 8.1 Artificial intelligence–enabled DDI prediction

Artificial intelligence (AI) and machine learning (ML) are transforming the detection and prediction of DDIs by facilitating scalable, data-driven methodologies. Advanced deep learning models, such as graph neural networks (GNNs) and transformer-based architectures like BERT-DDI, are capable of revealing intricate molecular interactions and identifying novel DDIs from extensive compound libraries and biomedical literature. Additionally, natural language processing (NLP) techniques enhance the analysis of electronic health records (EHRs) and adverse event reports, allowing for the identification of hidden interaction signals. Nevertheless, for these technologies to gain wider regulatory acceptance, there is a need for improvements in model interpretability, clinical validation, and effective risk communication.

### 8.2 Network pharmacology and multi-DDI risk modeling

The increasing occurrence of polypharmacy necessitates the implementation of systems-level strategies. Tools such as network pharmacology models, agent-based simulations, and cumulative DDIs risk scores allow researchers to assess multi-drug treatment plans in a comprehensive manner. These methodologies are particularly significant in fields like geriatrics, oncology, and psychiatry, where the simultaneous administration of various medications is frequently observed.

### 8.3 Beyond CYP450: epigenetics, microbiome, and immune modulation

DDIs mechanisms are evolving beyond traditional pathways, introducing new complexities such as epigenetic modulation, microbiome-driven metabolism, and immune-mediated enzyme regulation. For instance, histone deacetylase inhibitors have been shown to influence the expression of CYP3A4, an important enzyme in drug metabolism. Additionally, the gut microbiota plays a significant role in drug metabolism, as it can either activate or inactivate certain medications and alter the host’s metabolic capacity. These emerging mechanisms highlight the need for their incorporation into existing DDIs prediction frameworks to enhance our understanding and management of drug interactions.

### 8.4 Pharmacogenomics and genotype-guided decision support

Genetic polymorphisms play a significant role in influencing the drug behaviors of both perpetrators and victims. New models that focus on gene–drug–drug interactions (GDDIs) are being developed alongside electronic health record (EHR)-integrated CDSS to provide alerts tailored to specific genotypes. By incorporating polygenic risk scores and dynamic enzyme activity models, these systems may improve the assessment of risks associated with drug interactions. Pharmacogenomics is essential for personalizing treatment, particularly in the realm of psychiatric medications, where it can help prevent drug intolerance. For example, pharmacogenetic testing has been shown to reduce the risk of adverse drug reactions in patients prescribed antipsychotics, as highlighted in a specific case report (Hudnik et al., 2024).

### 8.5 Innovative study designs and simulation-guided trials

To improve study efficiency and ensure relevance to real-world applications, innovative methodologies like adaptive trials, microdosing studies, and physiologically based pharmacokinetic (PBPK)-informed trial designs are becoming more popular. Regulatory agencies are increasingly supporting simulation-based strategies as an integral component of model-informed drug development (MIDD).

### 8.6 Global harmonization and open science

The adoption of ICH M12 and the establishment of open-access DDIs databases, such as ONC-DDI and FDA DDIR, signify a significant shift towards harmonized regulatory science and enhanced transparency in the field. These initiatives are supported by collaborative consortia that are actively working to set standards, promote data sharing, and train artificial intelligence models. This collaborative effort is crucial in accelerating global advancements in DDIs safety, ensuring that stakeholders can access and utilize vital information effectively.

## 9 Conclusion

DDIs present a complex challenge in modern pharmacotherapy, particularly in an era characterized by polypharmacy, biologics, and precision medicine. As treatment plans become increasingly intricate, it is crucial to identify and address DDIs promptly to ensure both the effectiveness of therapies and the safety of patients.

This review offers a thorough overview of the current landscape of DDIs, covering the underlying mechanisms, methods for computational prediction, considerations specific to different populations, and the evolving regulatory frameworks. By integrating traditional pharmacological principles with advanced technologies like artificial intelligence (AI), real-world data (RWD), and pharmacogenomics, we highlight the critical role of interdisciplinary collaboration in developing the next-generation of DDIs management.

AI-powered models, knowledge graphs, and NLP-based pharmacovigilance systems are increasingly revealing previously unrecognized interactions that are sensitive to context, and they are doing so with greater precision. However, to achieve successful clinical implementation, it is essential to systematically tackle several translational challenges. These include improving model interpretability, addressing data heterogeneity, and ensuring a diverse population representation in the data used.

Special populations, such as the elderly, children, pregnant individuals, and those with unique genetic profiles affecting drug metabolism, require customized strategies because their bodies process medications differently, and they are often underrepresented in clinical trials. Additionally, the increasing use of herbal and complementary medicines, combined with the diverse prescribing practices observed worldwide, highlights the need for DDIs approaches that are both culturally sensitive and centered around the patient’s individual needs.

Looking ahead, the field is experiencing a significant transformation, moving away from traditional static assessments of DDIs that typically consider only pairwise relationships. Instead, there is a shift towards dynamic models that are tailored to individual patients. This evolution is being driven by advancements in explainable artificial intelligence, which helps clarify how AI systems make decisions, as well as genotype-guided therapy, which personalizes treatment based on a patient’s genetic makeup. Additionally, the design of clinical trials is becoming more informed by simulations, allowing for a better understanding of how different treatments may interact in real-world scenarios. To successfully integrate these innovations into everyday medical practice, it will be essential to adopt interoperable decision support systems and maintain open-access databases for DDIs. These tools will enable healthcare providers to offer more personalized, responsive, and context-aware risk mitigation strategies for their patients.

In conclusion, research on DDIs is advancing beyond traditional pharmacological limits, transforming into a genuinely multidisciplinary effort. By integrating technological advancements, proactive regulatory strategies, and a focus on patient-centered design, this field is set to provide safer, more intelligent, and globally equitable pharmacotherapy in the future.

The convergence of artificial intelligence (AI), clinical pharmacology, and regulatory science, as shown in Figure 1, is transforming the detection, interpretation, and application of DDIs to enhance therapeutic safety. Implementing this multidimensional framework in practical, real-world environments marks the next significant advancement in personalized pharmacovigilance.

## References

[B1] Abdu N. Idrisnur S. Said H. Kifle L. Habte N. Ghirmai S. (2025). Inappropriate medication prescribing, polypharmacy, potential drug-drug interactions and medication regimen complexity in older adults attending three referral hospitals in Asmara, Eritrea: a cross-sectional study. BMC Geriatrics 25, 76. 10.1186/s12877-025-05736-9 39901132 PMC11789384

[B2] Abdullahi T. Gemou I. Nayak N. V. Murtaza G. Bach S. H. Eickhoff C. (2025). K-Paths: reasoning over graph paths for drug repurposing and drug interaction prediction. arXiv. 10.48550/arXiv.2502.13344arXiv

[B3] Ahmed A. Tanveer M. Dujaili J. A. Chuah L. H. Hashmi F. K. Awaisu A. (2023). Pharmacist-involved antiretroviral stewardship programs in people living with HIV/AIDS: a systematic review. AIDS Patient Care STDs 37 (1), 31–52. 10.1089/apc.2022.0192 36626156

[B107] Alemayehu T. T. Wassie Y. A. Bekalu A. F. Tegegne A. A. Ayenew W. Tadesse G. (2024). Prevalence of potential drug-drug interactions and associated factors among elderly patients in Ethiopia: a systematic review and meta-analysis. Glob. Health Res. Policy 9 (46), 1–13. 10.1186/s41256-024-00386-7 39533381 PMC11559191

[B4] Al-Rabeah M. H. Lakizadeh A. (2022). Prediction of drug-drug interaction events using graph neural networks based feature extraction. Sci. Rep. 12, 15590. 10.1038/s41598-022-19999-4 36114278 PMC9481536

[B5] Albogami Y. Alfakhri A. Alaqil A. Alkoraishi A. Alshammari H. Elsharawy Y. (2024). Safety and quality of AI chatbots for drug-related inquiries: a real-world comparison with licensed pharmacists. Digit. Health 10, 20552076241253523. 10.1177/20552076241253523 38757086 PMC11097738

[B6] AlHarkan K. S. Alsousi S. AlMishqab M. Alawami M. Almearaj J. Alhashim H. (2023). Associations between polypharmacy and potentially inappropriate medications with risk of falls among the elderly in Saudi Arabia. BMC Geriatr. 23 (1), 222. 10.1186/s12877-023-03852-y 37024805 PMC10080807

[B7] Alhussain K. Al Dandan A. Al Elaiwi H. Al W. H. Al Abdulathim A. Almohaish S. (2024). Factors associated with the practice of assessing drug-drug interactions among pharmacists in Saudi Arabia. Healthcare 12 (22), 2285. 10.3390/healthcare12222285 39595482 PMC11594466

[B8] Almodovar A. S. Keller M. Lee J. H. Mehta H. B. Manja V. Nguyen T. P. P. (2024). Deprescribing medications among patients with multiple prescribers: a socioecological model. J. Am. Geriatr. Soc. 72 (3), 660–669. 10.1111/jgs.18667 37943070 PMC10947820

[B9] Alsanosi S. M. Padmanabhan S. (2024). Potential applications of artificial intelligence (AI) in managing polypharmacy in Saudi Arabia: a narrative review. Healthcar 12 (7), 788. 10.3390/healthcare12070788 PMC11011812 38610210

[B10] Andrade A. Nascimento T. Cabrita C. Leitao H. Pinto E. (2024). Potentially inappropriate medication: a pilot study in institutionalized older adults. Healthcare 12 (13), 1275. 10.3390/healthcare12131275 38998810 PMC11241476

[B11] Anrys P. Petit A. E. Thevelin S. Sallevelt B. Drenth C. Soiza R. L. (2021). An international consensus list of potentially clinically significant drug-drug interactions in older people. J. Am. Med. Direct. Assoc. 22 (4), 2121–2133.e24. 10.1016/j.jamda.2021.03.019 33901428

[B12] Baptista T. Serrano A. Lo Presti A. P. Fernandez-Arana A. Elkis H. Motuca M. (2024). Clozapine safety monitoring and related research in psychiatry and neurology in South America: a scoping review. Schizophrenia Res. 268, 29–33. 10.1016/j.schres.2023.07.029 37541864

[B13] Berk M. Köhler-Forsberg O. Turner M. Penninx B. W. J. H. Wrobel A. Firth J. (2023). Comorbidity between major depressive disorder and physical diseases: a comprehensive review of epidemiology, mechanisms and management. World Psychiatry 22 (3), 366–387. 10.1002/wps.21110 37713568 PMC10503929

[B14] Boehnke K. F. Cox K. Weston C. Herberholz M. Glynos N. Kolbman N. (2023). Slouching towards engagement: interactions between people using psychedelics naturalistically and their healthcare providers. Front. Psych. 14, 1224551. 10.3389/fpsyt.2023.1224551 PMC10436225 37599880

[B15] Brown J. D. Rivera K. J. R. Hernandez L. Y. C. Doenges M. R. Auchey I. Pham T. (2021). Natural and synthetic cannabinoids: pharmacology, uses, adverse drug events, and drug interactions. J. Clin. Pharmacol. 61 (S1), S37–S52. 10.1002/jcph.1871 34396558

[B16] Buick J. K. Williams A. Meier M. J. Swartz C. D. Recio L. Gagné R. (2021). A modern genotoxicity testing paradigm: integration of the high-throughput CometChip® and the TGx-DDI transcriptomic biomarker in human HepaRG™ cell cultures. Front. Public Health 9, 694834. 10.3389/fpubh.2021.694834 34485225 PMC8416458

[B17] Büyükkasap A. E. Yazici G. (2024). Knowledge levels of doctors and nurses working in surgical clinics about nutrients and food supplements, a multicentre descriptive study. BMC Nursing 23 (1), 277. 10.1186/s12912-024-01968-z 38664695 PMC11044485

[B18] Carollo M. Crisafulli S. Vitturi G. Besco M. Hinek D. Sartorio A. (2024). Clinical impact of medication review and deprescribing in older inpatients: a systematic review and meta-analysis. J. Am. Geriatr. Soc. 72 (10), 3219–3238. 10.1111/jgs.19035 38822740

[B19] Chastain D. B. Curtis J. Tang E. M. Y. Young H. N. Ladak A. F. (2024). ART-related medication errors in hospitalized people with HIV in the INSTI-era: analysis from 2 health systems in South Georgia, US. AIDS Care 36 (6), 832–839. 10.1080/09540121.2023.2248564 37614179

[B20] Chen Y. Wang J. Zou Q. Niu M. Ding Y. Song J. (2024). DrugDAGT: a dual-attention graph transformer with contrastive learning improves drug-drug interaction prediction. BMC Biol. 22 (233), 233. 10.1186/s12915-024-02030-9 39396972 PMC11472440

[B21] Cirrincione L. R. Huang K. J. (2021). Sex and gender differences in clinical pharmacology: implications for transgender medicine. Clin. Pharmacol. Therapeut. 110 (6), 1360–1374. 10.1002/cpt.2330 PMC8518665 33763856

[B22] Coroa M. C. P. Mendes P. F. S. Baia-da-Silva D. C. Souza-Monteiro D. Ferreira M. K. M. Braga G. L. C. (2023). What is known about midazolam? A bibliometric approach of the literature. Healthcare 11 (1), 96. 10.3390/healthcare11010096 PMC9819597 36611556

[B23] Cuomo A. Aguglia A. De Berardis D. Ventriglio A. Gesi C. Fagiolini A. (2024). Individualized strategies for depression: narrative review of clinical profiles responsive to vortioxetine. Ann. Gener. Psychiatry 23 (1), 20. 10.1186/s12991-024-00505-1 PMC11097484 38755657

[B24] Danic M. Markovic N. Ostojic T. Kojic M. Lazarević S. Mikov M. (2024). Intestinal microbiota, probiotics and their interactions with drugs: knowledge, attitudes and practices of health science students in Serbia. BMC Med. Educat. 24 (1), 1381. 10.1186/s12909-024-06249-6 PMC11600795 39605036

[B25] Dwivedi J. Kaushal S. Wal P. Jogi Chandrashekhar D. Sharma A. Nathiya D. (2025). A data mining approach on polypharmacy and drug-drug interactions of common diabetes medications. Curr. Drug Metabol. 26 (1), 12–29. 10.2174/0113892002358291250401190533 40248924

[B26] Ekpor E. Osei E. Akyirem S. (2024). Prevalence and predictors of traditional medicine use among persons with diabetes in Africa: a systematic review. Inter. Health 16 (3), 252–260. 10.1093/inthealth/ihad080 PMC11062204 37706354

[B27] Fiorillo A. Sampogna G. Albert U. Maina G. Perugi G. Pompili M. (2023). Facts and myths about the use of lithium for bipolar disorder in routine clinical practice: an expert consensus paper. Ann. Gener. Psychiatry 22 (1), 50. 10.1186/s12991-023-00481-y PMC10702081 38057894

[B28] Forte M. d'Amati A. Limongelli L. Corsalini M. Favia G. Ingravallo G. (2024). Could MRONJ be related to osimertinib monotherapy in lung cancer patients after denosumab suspension? Healthcare 12 (4), 457. 10.3390/healthcare12040457 38391832 PMC10888159

[B29] Fravel M. A. Ernst M. E. Gilmartin-Thomas J. Woods R. L. Orchard S. G. Owen A. J. (2023). Dietary supplement and complementary and alternative medicine use among older adults in Australia and the United States. J. Am. Geriatr. Soc. 71 (7), 2219–2228. 10.1111/jgs.18305 36852896 PMC10460828

[B30] Gao J. Wu Z. Al-Sabri R. Oloulade B. M. Chen J. (2024). AutoDDI: drug–drug interaction prediction with automated graph neural network. IEEE J. Biomed. Health Informat. 28 (2), 1773–1784. 10.1109/jbhi.2024.3349570 38446657

[B31] Gentile G. Casale A. D. De Luca O. Salerno G. Spirito S. Regiani M. (2024). Recognizing and preventing unacknowledged prescribing errors associated with polypharmacy. Arch. Public Health 82 (1), 146. 10.1186/s13690-024-01381-7 39232813 PMC11373128

[B32] Gillman P. K. van den Eynde V. Godet L. Redhead C. Horwitz A. Barnett B. (2023). Monoamine oxidase inhibitors and clinically relevant drug interactions: a guide for preventing serotonin toxicity and hypertensive reactions. Psychiatry Annal. 53 (8), 353–358. 10.3928/00485713-20230713-02

[B33] Grammatikopoulou M. Zachariadou M. Zande M. Giannios G. Chytas A. Karanikas H. (2024a). Evaluation of an electronic prescription platform: clinicians’ feedback on three distinct services aiming to facilitate clinical decision and safer e-prescription. Res. Soc. Administr. Pharm. 20 (7), 640–647. 10.1016/j.sapharm.2024.04.004 38653646

[B34] Grammatikopoulou M. Lazarou I. Giannios G. Kakalou C. A. Zachariadou M. Zande M. (2024b). Electronic prescription systems in Greece: a large-scale survey of healthcare professionals’ perceptions. Arch. Public Health 82 (1), 68. 10.1186/s13690-024-01304-6 38730501 PMC11088065

[B35] Güvel M. C. Borazan F. Y. Varan H. D. Göker B. Uluoğlu C. (2024). Detection of potentially inappropriate prescriptions using TIME and STOPP/START lists in Turkish geriatric patients: a single center experience. Turk. J. Geriatr. 27 (4), 339–348. 10.29400/tjgeri.2024.407

[B36] Hart L. A. Vo S. Hanlon J. T. Schmader K. E. Gray S. L. (2025). Medication use quality and safety in older adults: 2023 update. J. Am. Geriatr. Soc. 73, 1704–1710. 10.1111/jgs.19360 39868607 PMC12215870

[B37] Heck J. zu Siederdissen C. H. Krause O. Schröder S. Schulze Westhoff M. Strunz P. P. (2024). Concordance of emergency department physicians’ decisions on HIV post-exposure prophylaxis with national guidelines: results from a retrospective cohort study. Inter. Health 16 (2), 219–226. 10.1093/inthealth/ihad076 PMC10911529 37624102

[B38] Hire A. J. Franklin B. D. (2024). Potentially inappropriate prescribing (PIP) in older people and its association with socioeconomic deprivation-a systematic review and narrative synthesis. BMC Geriatr. 24 (1), 651–11. 10.1186/s12877-024-04858-w 39095729 PMC11295679

[B39] Ho L. A. Kwong M. H. Li A. S. C. Nilsen P. Ho F. F. Zhong C. C. W. (2024). Developing implementation strategies for promoting integrative oncology outpatient service delivery and utilisation: a qualitative study in Hong Kong. Front. Public Health 12, 1414297. 10.3389/fpubh.2024.1414297 39281081 PMC11392861

[B40] Hochheiser H. Jing X. Garcia E. A. Ayvaz S. Sahay R. Dumontier M. (2021). A minimal information model for potential drug-drug interactions. Front. Pharmacol. 12, 608068. 10.3389/fphar.2020.608068 PMC7982727 33762928

[B41] Hu J. Bao R. Lin Y. Zhang H. Xiang Y. (2024). Accurate medical named entity recognition through specialized NLP models. arXiv. 10.48550/arXiv.2412.08255arXiv

[B42] Huang A. Xie X. Yao X. Liu H. Wang X. Peng S. (2023). HF-DDI: predicting drug-drug interaction events based on multimodal hybrid fusion. J. Computat. Biol. 30 (9), 961–971. 10.1089/cmb.2023.0068 37594774

[B43] Hudnik L. K. Blagus T. Trampuz S. R. Dolzan V. Bon J. Pjevac M. (2024). Case report: avoiding intolerance to antipsychotics through a personalized treatment approach based on pharmacogenetics. Front. Psychiatry 15, 1363051. 10.3389/fpsyt.2024.1363051 38566958 PMC10985247

[B44] Inglis J. M. Caughey G. Thynne T. Brotherton K. Liew D. Mangoni A. A. (2024). Inappropriate prescribing and association with readmission or mortality in hospitalised older adults with frailty: a systematic review and meta-analysis. BMC Geriatr. 24 (1), 718. 10.1186/s12877-024-05297-3 39210280 PMC11363439

[B45] Inoue Y. Song T. Wang X. Luna A. Fu T. (2025). DrugAgent: multi-agent large language model-based reasoning for drug-target interaction prediction. arXiv, arXiv:2408.13378v4. 10.48550/arXiv.2408.13378

[B46] Kaur U. Chakrabarti S. S. Gupta G. K. Singh A. Gambhir I. S. (2024). Drug-related problems in older adults in outpatient settings: results from a 6-year long prospective study in a tertiary hospital of North India. Geriatr. Gerontol. Inter. 24, 285–291. 10.1111/ggi.14650 37577765

[B47] Kawakami Y. Matsuda T. Hidaka N. Tanaka M. Kimura E. (2024). Toward a unified understanding of drug-drug interactions: mapping Japanese drug codes to RxNorm concepts. J. Am. Med. Informat. Assoc. 31 (7), 1561–1568. 10.1093/jamia/ocae094 PMC11187495 38758661

[B48] Keenan A. Whichello C. Le H. H. Kern D. M. Fernandez G. S. Turner V. (2024). Patients' preferences for Sphingosine-1-phosphate receptor modulators in multiple sclerosis based on clinical management considerations: a choice experiment. Patient-Patient-Center. Outcomes Res. 17 (6), 685–696. 10.1007/s40271-024-00699-2 38748388

[B49] Kern L. M. Riffin C. Phongtankuel V. Aucapina J. E. Banerjee S. Ringel J. B. (2024). Gaps in the coordination of care for people living with dementia. J. Am. Geriatr. Soc. 72 (10), 3119–3128. 10.1111/jgs.19105 39073783 PMC11461100

[B50] Kim H. H. Goetz T. G. Grieve V. Keuroghlian A. S. (2023). Psychopharmacological considerations for gender-affirming hormone therapy. Harv. Rev. Psychiatry 31 (4), 183–194. 10.1097/HRP.0000000000000373 37437250 PMC10348476

[B51] Kriner P. Brieger P. Pogarell O. Schüle C. Mußmann L. Korbmacher J. (2024). Treatment of bipolar depression: clinical practice vs. adherence to guidelines-data from a Bavarian drug surveillance project. Front. Psychiatry 15, 1425549. 10.3389/fpsyt.2024.1425549 39015883 PMC11250482

[B52] Kupisz-Urbanska M. Pludowski P. Marcinowska-Suchowierska E. Marrone G. Cantelmo M. Cardillo C. (2021). Gut dysbiosis and Western diet in the pathogenesis of essential arterial hypertension: a narrative review. Nutrients 13 (4), 1162. 10.3390/nu13041162 33915885 PMC8066853

[B53] Leslie C. R. (2024). Pharmacy deserts and antitrust law. Bost. Univ. Law Rev. 104 (6), 1593–1655. Available online at: https://www.bu.edu/bulawreview/files/2024/12/LESLIE.pdf.

[B54] Li Y. Liu H. Chen X. Zhao Y. Gao T. Dong H. (2023). DDI-GCN: drug-drug interaction prediction *via* explainable graph convolutional networks. Artif. Intell. Med. 144, 102640. 10.1016/j.artmed.2023.102640 37783544

[B55] Liu G. Zhang Y. Liu X. Yao Q. (2025). Case-based reasoning enhances the predictive power of large language models in drug-drug interaction prediction. arXiv. 10.48550/arXiv.2505.23034arXiv

[B56] Ma M. Lei X. (2023). A dual graph neural network for drug–drug interactions prediction based on molecular structure and interactions. PLoS Computat. Biol. 155, 106602. 10.1016/j.compbiomed.2023.106602 PMC9879511 36701288

[B57] Martin-Oliveros A. Zamora J. P. Monaco A. Iriarte J. A. (2024). Multidose drug dispensing in community healthcare settings for patients with multimorbidity and polypharmacy. Inquiry J. Healthcare Prov. Public Health 61, 469580241274268. 10.1177/00469580241274268 PMC11526267 39373170

[B58] Martins M. F. L. Heydari P. Li W. L. Martinez-Chávez A. Venekamp N. Lebre M. C. (2022). Drug transporters ABCB1 (P-gp) and OATP, but not drug-metabolizing enzyme CYP3A4, affect the pharmacokinetics of the psychoactive alkaloid ibogaine and its metabolites. Front. Pharmacol. 13, 878165. 10.3389/fphar.2022.878165 PMC8931498 35308219

[B59] Moon J. Chladek J. S. Wilson P. Chui M. A. (2024). Clinical decision support systems in community pharmacies: a scoping review. J. Am. Med. Informat. Assoc. 31 (1), 231–239. 10.1093/jamia/ocad208 PMC10746304 37875066

[B60] Mubaslat O. Zhang V. Z. Moles R. (2024). Improving the medication literacy at the time of discharge from hospital (the LiMeTiD study). Res. Soc. Administr. Pharm. 20 (12), 1125–1133. 10.1016/j.sapharm.2024.09.003 39306513

[B61] Muylle K. M. Gentens K. Dupont A. G. Cornu P. (2021). Evaluation of an optimized context-aware clinical decision support system for drug-drug interaction screening. Internat. J. Med. Informat. 150, 104458. 10.1016/j.ijmedinf.2021.104458 33486355

[B62] Oliveira R. F. Oliveira A. I. Cruz A. S. Ribeiro O. Afreixo V. Pimentel F. (2024). Polypharmacy and drug interactions in older patients with cancer receiving chemotherapy: associated factors. BMC Geriatr. 24 (1), 557–14. 10.1186/s12877-024-05135-6 38918696 PMC11201315

[B63] Parmar A. Pal A. (2024). Advocating for the inclusion of therapeutic drug monitoring in the national essential diagnostic list: perspectives from psychiatrists. Indian J. Psychiatry 66 (7), 660–664. 10.4103/indianjpsychiatry.indianjpsychiatry_330_24 39257509 PMC11382748

[B64] Patel S. Tareen K. Patel C. Rosinski A. (2024). Herbal and non-herbal dietary supplements for psychiatric indications: considerations in liver transplantation. Curr. Psychiatry Rep. 26 (8), 436–446. 10.1007/s11920-024-01517-0 38941032

[B65] Perdixi E. Ramusino M. C. Costa A. Bernini S. (2024). Polypharmacy, drug-drug interactions, anticholinergic burden and cognitive outcomes: a snapshot from a community-dwelling sample of older men and women in northern Italy. Eur. J. Ageing 21 (1), 1–14. 10.1007/s10433-024-00806-0 38551689 PMC10980670

[B66] Qi Z. Wei Y. Liu L. (2025). A domain adaptive interpretable substructure-aware graph attention network for drug–drug interaction prediction. Interdiscip. Sci. 17 (3), 379–391. 10.1007/s12539-024-00680-5 39775539

[B67] Ruan C. J. Olmos I. Ricciardi C. Schoretsanitis G. Vincent P. D. Anıl Yağcıoğlu A. E. (2024). Exploring low clozapine C/D ratios, inverted clozapine-norclozapine ratios and undetectable concentrations as measures of non-adherence in clozapine patients A literature review and a case series of 17 patients from 3 studies. Schizophrenia Re. 268, 293–301. 10.1016/j.schres.2023.07.002 37487869

[B68] Schneider J. Algharably E. A. Budnick A. Wenzel A. Dräger D. Kreutz R. (2021). High prevalence of multimorbidity and polypharmacy in elderly patients with chronic pain receiving home care are associated with multiple medication-related problems. Front. Pharmacol. 12, 686990. 10.3389/fphar.2021.686990 34168565 PMC8217758

[B69] Sharma A. E. Tran A. S. Dy M. Najmabadi A. L. Olazo K. Huang B. T. C. (2024). Patient and caregiver perspectives on causes and prevention of ambulatory adverse events: multilingual qualitative study. BMJ Qual. Safe. 34, 507–519. 10.1136/bmjqs-2023-016955 38991703

[B70] Sheikh-Taha M. Asmar M. (2021). Polypharmacy and severe potential drug-drug interactions among older adults with cardiovascular disease in the United States. BMC Geriatr. 21 (1), 233. 10.1186/s12877-021-02183-0 33827442 PMC8028718

[B71] Sinaci A. A. Gencturk M. Alvarez-Romero C. (2024). Privacy-preserving federated machine learning on FAIR health data: a real-world application. Computat. Struct. Biotechnol. J. 22, 1163–1171. 10.1016/j.csbj.2024.02.015 PMC10904920 38434250

[B72] Smith R. T. Gruber S. A. (2023). Contemplating cannabis? The complex relationship between cannabinoids and hepatic metabolism resulting in the potential for drug-drug interactions. Front. Psychiatry 13, 1055481. 10.3389/fpsyt.2022.1055481 36704740 PMC9871609

[B73] Stout J. A. Allamong M. Hung F. Link K. Chan C. Muiruri C. (2024). Engagement in care, awareness, and interest in long-acting injectable anti-retroviral therapy. AIDS Behavior 28 (10), 3315–3325. 10.1007/s10461-024-04423-x 38954172 PMC11427500

[B74] Strawn J. R. Poweleit E. A. Uppugunduri C. R. S. Ramsey L. B. (2021). Pediatric therapeutic drug monitoring for selective serotonin reuptake inhibitors. Front. Pharmacol. 12, 749692. 10.3389/fphar.2021.749692 34658889 PMC8517085

[B75] Sun J. Zheng H. (2025). HDN-DDI: a novel framework for predicting drug-drug interactions using hierarchical molecular graphs and enhanced dual-view representation learning. BMC Bioinformat. 26 (28), 28–20. 10.1186/s12859-025-06052-0 PMC11765940 39863877

[B76] Swinglehurst D. Hogger L. Fudge N. (2023). Negotiating the polypharmacy paradox: a video-reflexive ethnography study of polypharmacy and its practices in primary care. BMJ Qual. Saf. 32 (3), 150–159. 10.1136/bmjqs-2022-014963 PMC9985753 36854488

[B77] Tamir A. Yuan J.-S. (2024). KITE-DDI: a knowledge graph integrated transformer model for accurately predicting drug-drug interactions events from drug SMILES and biomedical knowledge graph. arXiv. 10.48550/arXiv.2412.05770arXiv

[B78] Tian S.-Yu Zhou Z. Su X. Li Y.-F. (2025). Rethinking evaluation for multi-label drug-drug interaction prediction. Front. Compu. Sci. 19 (9), 199358. 10.1007/s11704-024-41055-9

[B79] Torazzi A. Tedesco E. Ceccato S. Santin L. Campagnari S. Losso L. (2024). Safety and efficacy of CyTisine for smoking cessation in a hOSPital context (CITOSP): study protocol for a prospective observational study. Front. Public Health 12, 1350176. 10.3389/fpubh.2024.1350176 39403432 PMC11471529

[B80] U.S. Food and Drug Administration (FDA) (2020). Clinical drug interaction studies-cytochrome P450 Enzyme- and transporter-mediated drug interactions: guidance for industry. 10.54724/FDA.CYPP450.2020

[B81] Uskur T. Güven O. Tat M. (2024). Retrospective analysis of lithium treatment: examination of blood levels. Front. Psychiatry 15, 1414424–10. 10.3389/fpsyt.2024.1414424 39279810 PMC11392753

[B82] Vandenberg A. E. Hwang U. Das S. Genes N. Nyamu S. Richardson L. (2024). Scaling the EQUIPPED medication safety program: traditional and hub-and-spoke implementation models. J. Am. Geriatr. Soc. 72 (7), 2184–2194. 10.1111/jgs.18746 38259070

[B83] Vasiliu O. (2023). The pharmacogenetics of the new-generation antipsychotics - a scoping review focused on patients with severe psychiatric disorders. Front. Psychiatry 14, 1124796. 10.3389/fpsyt.2023.1124796 36873203 PMC9978195

[B84] Vefghi A. Rahmati Z. Akbari M. (2025). Drug-target interaction/affinity prediction: deep learning models and advances review. *arXiv preprint* arXiv2502.15346 196, 110438. 10.1016/j.compbiomed.2025.110438 40609289

[B85] Verdoux H. Quiles C. de Leon J. (2024a). Risks and benefits of clozapine and lithium co-prescribing: a systematic review and expert recommendations. Schizophrenia Res. 268, 233–242. 10.1016/j.schres.2023.03.032 37002013

[B86] Verdoux H. Quiles C. de Leon J. (2024b). Optimizing antidepressant and clozapine co-prescription in clinical practice: a systematic review and expert recommendations. Schizophrenia Res. 268, 243–251. 10.1016/j.schres.2023.10.003 37852856

[B87] Wang Y. Xiong Y. Wu X. Sun X. Zhang J. Zheng G. (2024). DDIPrompt: drug-drug interaction event prediction based on graph prompt learning. arXiv, 2431–2441. 10.1145/3627673.3679645

[B88] Wang J. Y. Wang X. F. Li W. (2024a). Research on potential adverse drug reaction forecasting based on SAO semantic structure. IEEE Transact. Eng. Manage. 71, 2535–2548. 10.1109/TEM.2022.3187989

[B89] Wang Y. Yang Z. Yao Q. (2024b). Accurate and interpretable drug-drug interaction prediction enabled by knowledge subgraph learning. Communicat. Med. 4 (59), 59–12. 10.1038/s43856-024-00486-y PMC10978847 38548835

[B90] Wang N.-N. Zhu B. Li X.-L. Liu S. Shi J. Y. Cao D. S. (2024c). Comprehensive review of drug-drug interaction prediction based on machine learning: current status, challenges, and opportunities. J. Chem. Informat. Model. 64 (1), 96–109. 10.1021/acs.jcim.3c01304 38132638

[B91] Wang X. Liu G. Zhu B. He J. Zheng H. Zhang H. (2025). Pre-trained language models and few-shot learning for medical entity extraction. arXiv. 10.48550/arXiv.2504.04385

[B92] Wolff J. Hefner G. Normann C. Kaier K. Binder H. Hiemke C. (2021). Polypharmacy and the risk of drug-drug interactions and potentially inappropriate medications in hospital psychiatry. Pharmacoepidemiol. Drug Saf. 30 (9), 1258–1268. 10.1002/pds.5310 34146372

[B93] Womack J. A. Leblanc M. M. Sager A. S. Zaets L. N. Maisto S. A. Garcia A. (2025). The feasibility and acceptability of a clinical pharmacist-delivered intervention to reduce bothersome health symptoms from polypharmacy and alcohol use and communicate risk among people with HIV: pilot study protocol. AIDS .Behavior 29 (2), 482–496. 10.1007/s10461-024-04533-6 39465468 PMC12323700

[B94] Wu Y. X. Evans E. Boniface S. Britton A. (2024). Do older adults drink alcohol whilst taking alcohol-interactive medication? Prevalence and ten-year mortality risk: findings from the UK Whitehall II cohort study. Addict. Res. Theory 33, 199–204. 10.1080/16066359.2024.2380835

[B95] Xu C. Bulusu K. C. Pan H. Elemento O. (2024). DDI-GPT: explainable prediction of drug-drug interactions using large language models enhanced with knowledge graphs. bioRxiv, 2024.12.06.627266. 10.1101/2024.12.06.627266

[B96] Yao R. Shen Z. Xu X. Ling G. Xiang R. Song T. (2024). Knowledge mapping of graph neural networks for drug discovery: a bibliometric and visualized analysis. Front. Pharmacol. 15, 1393415. 10.3389/fphar.2024.1393415 38799167 PMC11116974

[B97] Zare P. Poustchi H. Mohammadi Z. Mesgarpour B. Akbari M. Kamalipour A. (2024). Polypharmacy and medication usage patterns in hypertensive patients: findings from the Pars Cohort Study. Res. Soc. Administ. Pharm. 20 (11), 1038–1046. 10.1016/j.sapharm.2024.07.006 39098543

[B98] Zerah L. Henrard S. Wilting I. O'Mahony D. Rodondi N. Dalleur O. (2021). Prevalence of drug-drug interactions in older people before and after hospital admission: analysis from the OPERAM trial. BMC Geriatr. 21 (1), 571. 10.1186/s12877-021-02532-z 34663238 PMC8524798

[B99] Zhang Y. Deng Z. Xu X. Feng Y. Junliang S. (2023). Application of artificial intelligence in drug-drug interactions prediction: a review. J. Chem. Informat. Model. 64 (6), 2158–2173. 10.1021/acs.jcim.3c00582 37458400

[B100] Zhang C. Jiang L. Hu K. Zhang Y. J. Han J. Chen J. (2024). Drug-drug interaction and initial dosage optimization of aripiprazole in patients with schizophrenia based on population pharmacokinetics. Front. Psychiatry 15, 1377268. 10.3389/fpsyt.2024.1377268 38957736 PMC11217561

[B101] Zhang S. Yu C. Zhang C. (2025). SCATrans: semantic cross-attention transformer for drug-drug interaction predication through multimodal biomedical data. BMC Bioinform. 26 (157), 157. 10.1186/s12859-025-06165-6 PMC12153160 40495152

[B102] Zhao Y. Chen W. Xu Y. Wang T. Ma W. Duan H. (2024). MGDDI: a multi-scale graph neural networks for drug-drug interaction prediction. Methods 228, 22–29. 10.1016/j.ymeth.2024.05.010 38754712

[B103] Zhou C. Zhang X. Li J. Song J. Xiang W. (2024). RGDA-DDI: residual graph attention network and dual-attention based framework for drug-drug interaction prediction. arXiv. 10.48550/arXiv.2408.15310arXiv

[B104] Zhu J. J. Liu Y. G. Zhang Y. Li D. X. (2021). Attribute supervised probabilistic dependent matrix tri-factorization model for the prediction of adverse drug-drug interaction. IEEE J. Biomed. Health Inform. 25 (8), 2820–2832. 10.1109/jbhi.2020.3048059 33373310

[B105] Zhu F. Zhang Y. Chen L. Qin B. Xu R. (2024a). Learning to describe for predicting zero-shot drug-drug interactions. arXiv. 10.48550/arXiv.2403.08377

[B106] Zhu A. Kuhnly N. Chen L. Dulu A. O. (2024b). A case study of polypharmacy-induced serotonin syndrome in a cancer patient. J. Am. Assoc. Nurse Practit. 36 (12), 728–732. 10.1097/JXX.0000000000001048 PMC12265877 39051987

